# YTHDC1-mediated augmentation of miR-30d in repressing pancreatic tumorigenesis via attenuation of RUNX1-induced transcriptional activation of Warburg effect

**DOI:** 10.1038/s41418-021-00804-0

**Published:** 2021-05-21

**Authors:** Yichao Hou, Qingwei Zhang, Wenjing Pang, Lidan Hou, Yu Liang, Xu Han, Xiaoyu Luo, Ping Wang, Xintian Zhang, Lei Li, Xiangjun Meng

**Affiliations:** 1 Department of Gastroenterology, Shanghai Nineth People’s Hospital, School of Medicine, Shanghai Jiao Tong University, Shanghai, China; 2 Digestive Disease Research and Clinical Translation Center, Shanghai Jiaotong University, Shanghai, China; 3 Division of Gastroenterology and Hepatology, Key Laboratory Gastroenterology and Hepatology, Ministry of Health, State Key Laboratory for Oncogenes and Related Genes, Renji Hospital, School of Medicine, Shanghai Jiao Tong University, Shanghai Institute of Digestive Disease, Shanghai, China

**Keywords:** Oncogenes, Cancer metabolism

## Abstract

Pancreatic ductal adenocarcinoma (PDAC) is one of the most lethal human cancers. It thrives in a malnourished environment; however, little is known about the mechanisms by which PDAC cells actively promote aerobic glycolysis to maintain their metabolic needs. Gene Expression Omnibus (GEO) was used to identify differentially expressed miRNAs. The expression pattern of miR-30d in normal and PDAC tissues was studied by in situ hybridization. The role of miR-30d/RUNX1 in vitro and in vivo was evaluated by CCK8 assay and clonogenic formation as well as transwell experiment, subcutaneous xenograft model and liver metastasis model, respectively. Glucose uptake, ATP and lactate production were tested to study the regulatory effect of miR-30d/RUNX1 on aerobic glycolysis in PDAC cells. Quantitative real-time PCR, western blot, Chip assay, promoter luciferase activity, RIP, MeRIP, and RNA stability assay were used to explore the molecular mechanism of YTHDC1/miR-30d/RUNX1 in PDAC. Here, we discover that miR-30d expression was remarkably decreased in PDAC tissues and associated with good prognosis, contributed to the suppression of tumor growth and metastasis, and attenuation of Warburg effect. Mechanistically, the m^6^A reader YTHDC1 facilitated the biogenesis of mature miR-30d via m^6^A-mediated regulation of mRNA stability. Then, miR-30d inhibited aerobic glycolysis through regulating SLC2A1 and HK1 expression by directly targeting the transcription factor RUNX1, which bound to the promoters of the SLC2A1 and HK1 genes. Moreover, miR-30d was clinically inversely correlated with RUNX1, SLC2A1 and HK1, which function as adverse prognosis factors for overall survival in PDAC tissues. Overall, we demonstrated that miR-30d is a functional and clinical tumor-suppressive gene in PDAC. Our findings further uncover that miR-30d is a novel target for YTHDC1 through m^6^A modification, and miR-30d represses pancreatic tumorigenesis via suppressing aerobic glycolysis.

## Introduction

Pancreatic ductal adenocarcinoma (PDAC) is characterized by a high-glycolysis rate to ensure its survival due to hypovascularization and the desmoplastic reaction, creating a nutrient-poor and highly hypoxic microenvironment [1]. Glycolysis in PDAC supports the vigorous growth of tumor cells by generating large amounts of substrates and promoting invasion and migration via interaction of glycolytic enzymes and actin [2]. Furthermore, important enzymes and intermediates of glycolysis can regulate PDAC metastasis through participating in signaling transduction or epigenetic regulation related to epithelial-mesenchymal transition (EMT), angiogenesis and colonization [3].

MicroRNAs (MiRNAs) are a class of non-coding small RNAs that negatively regulate gene expression by targeting specific mRNAs and inducing their degradation or translational suppression [4, 5]. Several studies have confirmed the importance of miRNA-mediated gene regulation in PDAC development and pathology and in metabolic abnormalities [6–8]. Previously, roles for miRNAs were described in the proliferation and metastasis of a variety of tumors, including colorectal cancer, liver cancer, pancreatic cancer and lung cancer, linking aberrant expression of miRNAs with the highly proliferative, migratory, and invasive phenotype of cancer cells [9–12].

N6-methyladenosine (m^6^A) modification is the most abundant posttranscriptional internal mRNA modification in eukaryotes [13, 14]. This modification is reversible, and its biological effects are mostly mediated through ‘writer’, ‘eraser’ and ‘reader’ proteins [13, 15]. Notably, the fate and function of m^6^A-methylated RNAs are controlled mostly through readers, including the YT521-B homology (YTH) domain-containing proteins (YTHDF1-3, YTHDC1 and YTHDC2), which can directly or indirectly bind and read m^6^A sites on mRNA [16–18]. These readers affect mRNA fate by regulating pre-mRNA splicing, facilitating translation or regulating mRNA stability [16, 19, 20]. Importantly, nuclear reader YTHDC1 has been suggested to play multiple roles in regulating mRNA splicing by preferably recruiting a certain splicing factor, expediting mRNA export, and accelerating decay of certain transcripts [21–25]. However, the exact molecular mechanisms by which YTHDC1 recognizes and regulates the expression of its targets remain elusive in PDAC tumorigenesis.

Herein, we identified that miR-30d was a glycolysis-associated miRNA that inhibited PDAC malignant phenotypes and independently predicted patient survival outcomes. Additionally, METTL3/14 depletion significantly decreased the amount of pri-miR-30d modified by m^6^A. Crucially, we provide evidence showing YTHDC1 preferentially recognizes m^6^A-modified pri-miR-30d and promotes the decay of pri-miR-30d, thereby promoting the initiation of miR-30d biogenesis through antagonizing termination of miRNA biogenesis of MCPIP1. Thus, our data revealed the critical tumor-suppressive roles of YTHDC1/miR-30d axis in PDAC development, with biological, mechanistic, and clinical impact on human PDAC and glycolysis pathways.

## Results

### Elevated miR-30d expression correlates with good prognosis of patients with PDAC

**Fig. 1:**
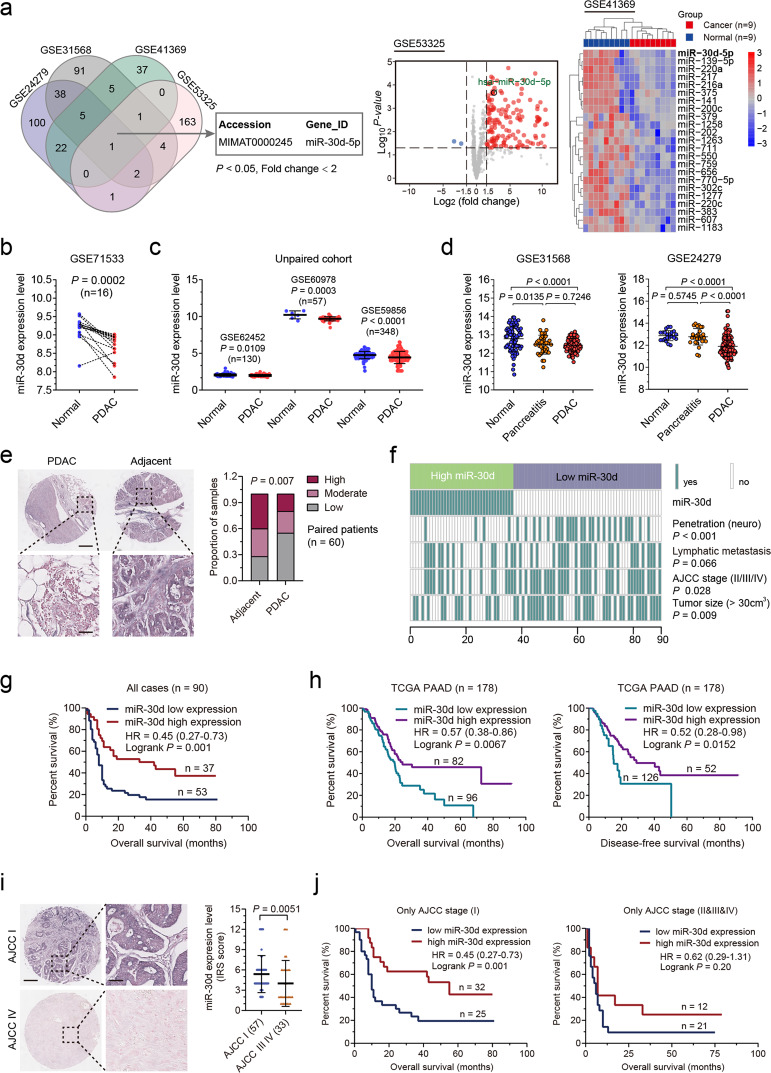
miR-30d expression is correlated with good prognosis in PDAC. **a** Left: Schematic illustration for screening for differentially expressed microRNAs (Fold change < 2, *P* < 0.05) using 4 independent cohorts (GSE24279, GSE31568, GSE41369, and GSE53325). Middle: Volcano plot showing fold changes (x axis) and corresponding *P* values (log_10_, y axis) of normal vs PDAC samples using GSE53325 dataset. Red dot represents fold change values >2 and *P* < 0.05. Right: Heatmap showing differentially expressed microRNAs between PDAC and normal pancreatic tissues. **b** Expression analysis of miR-30d in PDAC and matching normal pancreatic tissue samples in GEO dataset (GSE71533). **c** Expression analysis of miR-30d in PDAC and normal pancreatic tissue samples in 3 independent GEO datasets (GSE62452, GSE60987, and GSE59856). **d** Expression analysis of miR-30d in PDAC, pancreatitis, and normal pancreatic tissue samples in 2 independent GEO datasets (GSE31568, and GSE24279). **e** Representative in situ hybridization images of miR-30d in PDAC and paired adjacent tissues (left), and statistical analysis of proportion of high, moderate, and low staining in PDAC and paired adjacent tissues (right) in the PDAC TMA. Scale Bars, up: 200 μm; down: 50 μm. **f** Heatmap illustrating the association of different clinical characters with miR-30d high and low-expression tumors. **g** Kaplan–Meier analysis of overall survival for PDAC patients based on miR-30d expression in the PDAC TMA. **h** Kaplan–Meier analysis of overall survival (left) and disease-free survival (right) for PDAC patients based on miR-30d expression in the TCGA cohort. **i** Representative in situ hybridization images of miR-30d in AJCC stage I PDAC and AJCC stage IV PDAC (left), and statistical analysis of IRS score of miR-30d expression in pancreatic cancer tissues and paired adjacent tissues (right) in the PDAC TMA. Scale Bars, right: 200 μm; left: 50 μm. **j** Kaplan–Meier analysis of overall survival for PDAC patients based on miR-30d expression stratified by AJCC stage in the PDAC TMA.

To identify the potential miRNAs dysregulated in PDAC regardless of different races, we first analyzed the expression of miRNAs using GEO datasets from Western countries and Asian countries: GSE24279, GSE31568, GSE41369, and GSE53325. As shown in Fig. 1a, only miR-30d-5p was downregulated in cancer tissues compared with that in nontumor tissues (Fig. 1a), and the expression profile of miR-30d in PDAC was further confirmed in one paired PDAC cohort and three non-paired PDAC cohorts (Fig. 1b, c). However, the difference in the expression of miR-30d between pancreatitis and normal tissue or PDAC was not established (Fig. 1d).

We further performed in situ hybridization (ISH) staining on tissues from 90 patients with PDAC from the TMA, of which 60 had paired adjacent normal tissues. miR-30d expression was significantly lower in PDAC tissues than that in the adjacent tissues (Fig. 1e). We also found that miR-30d expression was negatively correlated with tumor size, penetration, lymphatic metastasis and AJCC II/III/IV stage (Fig. 1f, and Supplementary Table 2). Kaplan–Meier analysis revealed that high expression of the miR-30d was associated with a good prognosis in patients with PDAC (Fig. 1g). In addition, univariate or multivariate Cox regression analyses only identified positive association between good histological differentiation or high miR-30d expression and good overall survival (Supplementary Fig. 1a, b). Kaplan–Meier analysis using data derived from TCGA cohort also showed that PDAC patients with higher miR-30d expression had better overall survival and disease-free survival compared with those with lower miR-30d expression (Fig. 1h). Furthermore, miR-30d was more highly expressed in AJCC stage I PDAC than that in AJCC stage IV PDAC (Fig. 1i). Further Kaplan–Meier analysis stratified by AJCC stage showed that high miR-30d expression predicted good prognosis of patients with AJCC stage I PDAC but not patients with AJCC stage II/III/IV PDACs (Fig. 1j), indicating miR-30d high expression predicts good prognosis in early stage of PDAC patients.

### miR-30d suppresses tumor proliferation, metastasis, and angiogenesis both in vitro and in vivo

**Fig. 2:**
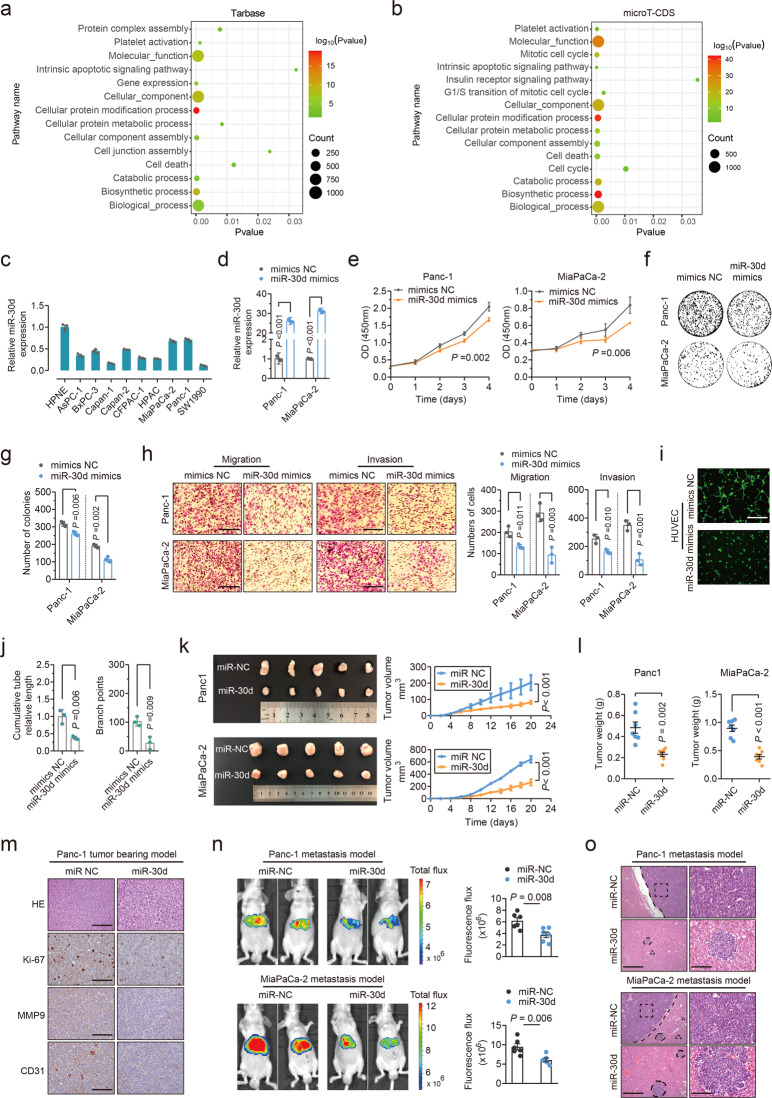
miR-30d suppresses tumor proliferation, metastasis, and angiogenesis both in vitro and in vivo. **a**, **b** GO Enrichment analysis (biological process) of miR-30d using DIANA tools based on public Tarbase and microT-CDS datasets. **c** qRT-PCR analysis of miR-30d mRNA expression in multiple PDAC cells. **d** miR-30d expression levels were evaluated using qRT–PCR in indicated cell lines with miR-30d mimics. **e** CCK8 assays were performed to determine the cell viability of Panc-1 and MiaPaCa-2 cell lines with miR-30d mimics transfection. **f**, **g** Colony formation assay was performed in two cell lines with miR-30d mimics transfection. **h** Transwell assays were performed to evaluate the effect of miR-30d on cell migration and invasion in Panc-1 and MiaPaCa-2 cells. Scale bars = 100 μm. **i**, **j** Capillary tube formation was performed to evaluate tumor angiogenesis in HUVECs with condition medium of Panc-1 cells with miR-30d mimics transfection. Scale bars = 100 μm. **K**, **l** Representative images of tumors with corresponding tumor volumes and tumor weights in nude mice bearing Panc-1 and MiaPaCa-2 cells with stable miR-30d overexpression or vector. **m** Representative immunohistochemical images of Ki-67, MMP9 and CD31 staining from miR-30d and vector subcutaneous xenograft tissues in Panc-1 tumor-bearing model. Scale bars = 100 μm. **n** Representative bioluminescent images of mice 8 weeks after splenic portal vein injection of Panc-1 and MiaPaCa-2 cells with miR-30d overexpression or vector-transfected cells and quantification of the results of bioluminescence imaging in the liver region. **o** Representative images and H&E staining of liver tissues from the above two groups are shown. Scale Bars, right: 200 μm; left: 50 μm.

To explore the role of miR-30d in PDAC progression, Gene Ontology (GO) and Kyoto Encyclopedia of Genes and Genomes (KEGG) analysis were performed using mirPath v.3. GO analysis results showed that miR-30d was greatly associated with cell death, cell cycle, cell junction assembly as well as cellular apoptotic signaling (Fig. 2a, b), and KEGG analysis results revealed that miR-30d was significantly associated with metabolic signals and major signaling pathways involved in tumor development (Supplementary Fig. 2a, b). Two cell lines with high miR-30d expression levels, Panc-1 and MiaPaCa-2, were selected for functional analysis (Fig. 2c). Functional experiment data showed that overexpression of miR-30d abolished cell proliferation (Fig. 2d, e) and colony formation (Fig. 2f, g) in two PDAC cell lines, while knockdown of miR-30d promoted cell proliferation (Supplementary Fig. 2c, d) and colony formation (Supplementary Fig. 2e, f). Ectopic expression of miR-30d also led to a significant increase in cell apoptosis and induced cell cycle arrest in the G0/G1 phase in both PDAC cell lines (Supplementary Fig. 2g, h). In vitro transwell assays demonstrated that cell migration and invasion were decreased in PDAC cells by miR-30d overexpression (Fig. 2h), and vice versa (Supplementary Fig. 2i). The tube formation assays show that the culture medium of miR-30d mimics transfected PDAC cells significantly inhibited capillary tube formation of HUVECs (Fig. 2i, j), and vice versa (Supplementary Fig. 2j). These results show that miR-30d affects cell proliferation, migration and angiogenesis in vitro.

Subcutaneous tumor xenografting assay in nude mice showed that the volume formed by PDAC cells stably expressing miR-30d was significantly smaller than that formed by control cells, with lighter tumor weight (Fig. 2k, l). Moreover, downregulation of Ki-67, MMP9 and CD31 were detected in miR-30d overexpressing xenografts than in controls (Fig. 2m, Supplementary Fig. 2k). Quantification of bioluminescence signaling showed that miR-30d overexpression attenuated the luminescence intensity and total flux in the livers (Fig. 2n) and dramatically suppressed PDAC liver metastasis (Fig. 2o). In summary, our in vivo results further support the tumor-suppressing role of miR-30d in PDAC.

### miR-30d suppresses glycolysis by inhibiting downstream SLC2A1 and HK1 via directly targeting RUNX1

**Fig. 3:**
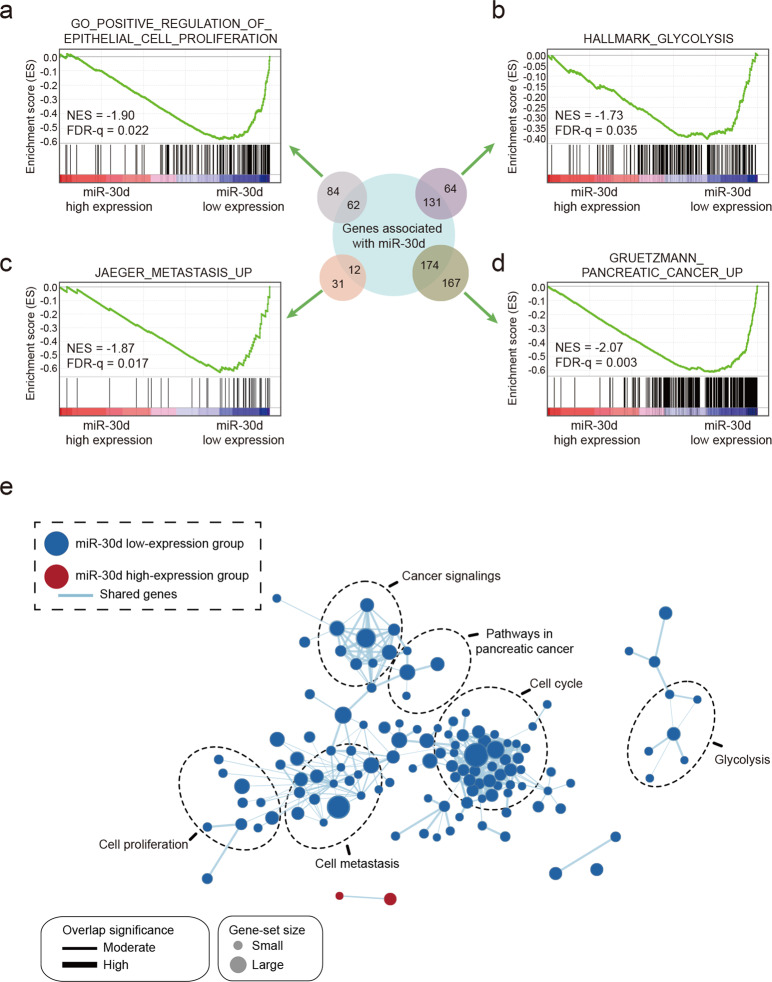
The correlation between tumorigenic gene sets and miR-30d expression levels is assessed via GSEA. **a–d** Overview of GSEA used to identify the differential gene profiles between PDAC with miR-30d high expression and PDAC with miR-30d low expression. **e** GSEA comparison between patients with high miR-30d expression (red) and patients with low miR-30d expression (blue). Different pathways and biological processes were elucidated between the two groups. Cytoscape and enrichment maps are used to visualize GSEA results. Nodes, representing enriched gene sets were grouped and annotated according to their similarity of the related gene sets. The enrichment results are mapped as a network of gene sets (nodes) with size proportional to the total number of genes in each gene set and the thickness of lines between nodes presenting the proportion of shared genes between genome. The enrichment map shows a set of activated genes related to glycolysis, cell proliferation, cell cycle, cancer signaling, and metastasis and pathways for PDAC in the enrichment map using the TCGA cohort. The enrichment score (ES, blue line) indicates the degree to which the gene set is overrepresented at the top or bottom of the ranked gene list. Black bars indicate the positions of genes belonging to the genome in the ranking list of genes included in the analysis.

To gain further insight into the biological pathways involved in pancreatic cancer pathogenesis, we performed Gene set enrichment analysis (GSEA) in the TCGA PAAD cohort based on the median of miR-30d expression levels. The results of GSEA revealed that the gene sets related to cell proliferation, metastasis, glycolysis, and pancreatic cancer-specific signature were negatively correlated with miR-30d high expression in PDACs (Fig. 3a–d), and gene signatures of cell proliferation, cell metastasis, cell cycle, cancer signaling, pathways in pancreatic cancer, and glycolysis were enriched in patients with low miR-30d expression, but not in patients with high miR-30d expression (Fig. 3e), implying that miR-30d may directly influence glycolytic metabolism to modulate pancreatic tumorigenesis. Indeed, both 2-deoxy-D-glucose (2-DG) and 3-bromopyruvate (3-BP) (two different inhibitors of glycolysis pathway) could significantly block miR-30d inhibitor-induced cell proliferation in vitro (Supplementary Fig. 3a, b) and in vivo (Supplementary Fig. 3c, d).

**Fig. 4:**
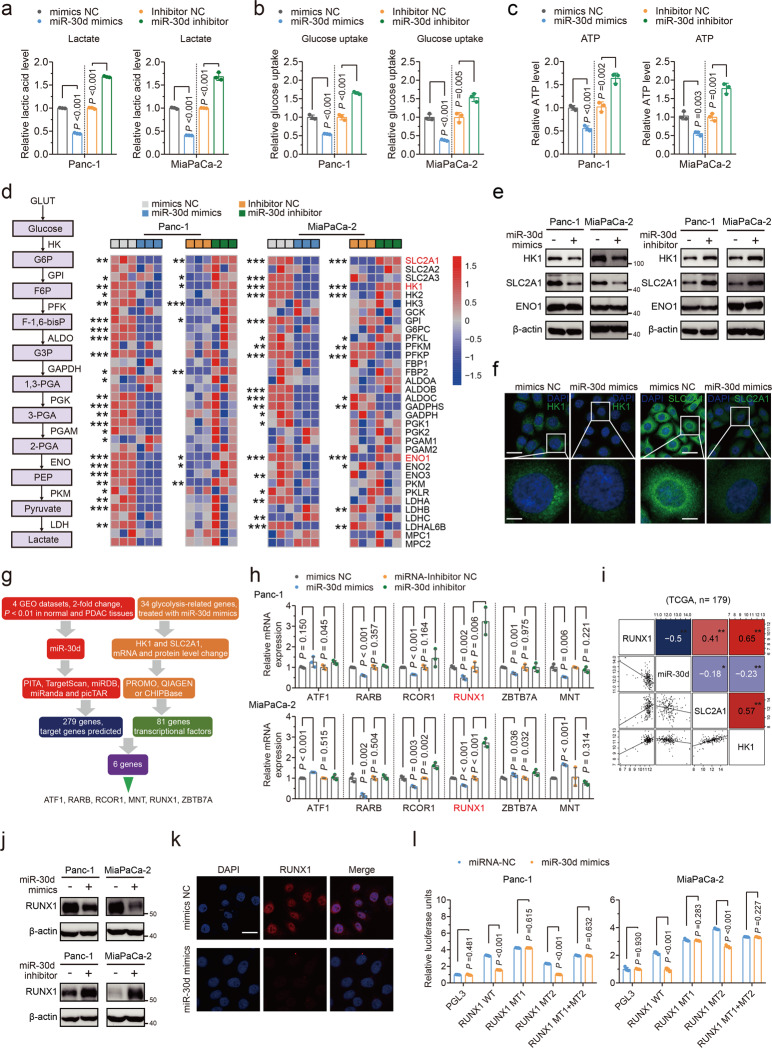
miR-30d suppresses glycolysis by inhibiting downstream SLC2A1 and HK1 via directly targeting RUNX1. Lactate production (**a**), glucose uptake (**b**) and ATP production (**c**) were measured by colorimetric analysis in two PDAC cells transfected with miR-30d mimics or miR-30d inhibitor. **d** mRNA expression levels of 34 aerobic glycolysis-related genes were evaluated using qRT–PCR in miR-30d mimics and miR-30d inhibitor transfected cell lines. **e** Protein expression levels of HK1, SLC2A1, and ENO1 were measured by western blot in two cell lines with miR-30d mimics and miR-30d inhibitor transfection. **f** Immunofluorescence staining of HK1 and SLC2A1 expression in Panc-1 cells with miR-30d mimics transfection. Scale Bars, up: 20 μm; down: 5 μm. **g** Schematic illustration of the protocol for screening transcription factors using PITA, TargetScan, miRDB, miRanda, and picTAR to predict miR-30d target genes and PROMO, QIAGEN, and CHIPBase to predict transcription factors targeting HK1 and SLC2A1. **h** mRNA expression levels of 5 transcriptional factors were evaluated using qRT–PCR in miR-30d mimics and miR-30d inhibitor transfected cell lines. **i** The correlation between miR-30d, RUNX1, SLC2A1 and HK1 expression of pancreatic tissues in the TCGA cohort. The square in the upper right corner demonstrates the Pearson correlation value between the indicated genes with blue indicating negative correlation and red indicating positive correlation. The square in the lower left corner shows the scatterplot matrix fitted trend line for indicated genes. **, correlation is significant at the 0.01 level; *, correlation is significant at the 0.05 level. **j**, **k** Protein expression levels of RUNX1 were measured by western blot and immunofluorescence staining in cells with indicated treatment. Scale bars = 20 μm. **l** Luciferase activity assays were performed to confirm the direct binding efficiency of miR-30d and its putative target RUNX1 with indicated treatment.

Additionally, overexpression of miR-30d greatly decreased glucose uptake and lactate secretion relative to control cells, and vice versa (Fig. 4a, b). Meanwhile, miR-30d also influenced intracellular ATP levels in PDAC cells (Fig. 4c). Correlation analyses using TCGA cohort showed that miR-30d was significantly negatively associated with multiple key glycolytic enzymes (Supplementary Fig. 4a). Importantly, miR-30d mimics induced the downregulation and miR-30d inhibitor induced the upregulation of SLC2A1, HK1 and ENO1 mRNA levels in both Panc-1 and MiaPaCa-2 cell lines (Fig. 4d). As a further confirmation, we found that only SLC2A1 and HK1 but not ENO1 protein levels were regulated by miR-30d, as detected with western blot (Fig. 4e), as well as by in situ intracellular HK1 and SLC2A1 staining (Fig. 4f).

No putative miR-30d binding sites were predicted in SLC2A1 and HK1. Previous studies have shown that transcription factors could regulate glycolysis [26, 27]. Here, we identified six transcription factors as the predictive miR-30d targeted transcription factors that may regulate SLC2A1 and HK1 (Fig. 4g), of which only RUNX1 was regulated by miR-30d at mRNA level (Fig. 4h). Of note, correlation analysis showed that RUNX1 mRNA expression was negatively associated with miR-30d and positively associated with aerobic glycolysis-related genes, including SLC2A1 and HK1 (Fig. 4i and Supplementary Fig. 4b). Similarly, RUNX1 protein was also regulated by miR-30d (Fig. 4j, k). There are two putative miR-30d binding sites in the RUNX1 3′UTR, and luciferase reporter assay showed that miR-30d or its inhibitor was able to regulate the luciferase activity of the WT reporter and its miR-30d binding site 2 mutant (MT2) but not site 1 mutant (MT1) (Supplementary Fig. 4c, d and Fig. 4l). Therefore, these results suggest miR-30d binds to the binding site 1 of the RUNX1 3′UTR.

It has been well documented that MYC [26] and HIF1α [27] regulate glycolysis pathways. Our results showed that miR-30d did not regulate their expression at mRNA or protein level (Supplementary Fig. 4e, f) and they did not regulate miR-30d (Supplementary Fig. 4g, h). Taken together, miR-30d suppresses glycolysis by inhibiting downstream SLC2A1 and HK1 via directly targeting RUNX1.

### miR-30d suppresses glycolysis via inhibiting RUNX1 binding to the promoter of SLC2A1 and HK1

**Fig. 5:**
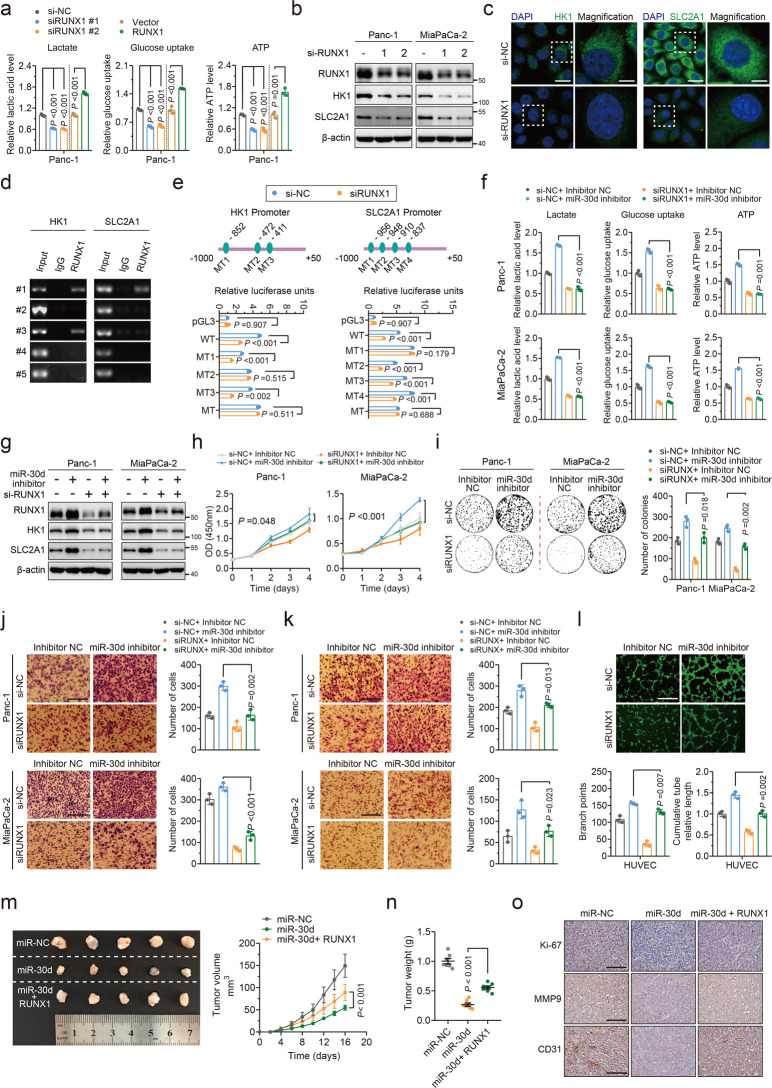
miR-30d suppresses glycolysis via inhibiting RUNX1 binding to the promoter of SLC2A1 and HK1. **a** Lactate production, glucose uptake and ATP production were measured by colorimetric analysis in Panc-1 cells with knockdown and overexpression of RUNX1. **b** Protein expression levels of RUNX1, HK1, and SLC2A1 were measured by western blot in two cell lines with knockdown of RUNX1. **c** Immunofluorescence staining of HK1 and SLC2A1 expression in Panc-1 with knockdown of RUNX1. Scale Bars, up: 20 μm; down: 5 μm. **d** PCR amplification of RUNX1-binging fragments following ChIP using an antibody against RUNX1 in Panc-1 cell lysates. #1, −1000~−791; #2, −790~−581; #3, −580~−371; #4, −370~161; #5, −160~+50. **e** Upper: Schematic diagram of wild-type (WT) and deletion mutants (MT) of HK1 (left) and SLC2A1 (right) promoter-containing fragments with putative RUNX1-binding sites. Lower: Luciferase activity in Panc-1 cells with RUNX1 knockdown following transfection with HK1 or SLC2A1 promoter luciferase reporter vectors. **f** Lactate production, glucose uptake and ATP production were measured by colorimetric analysis after transfection with miR-30d inhibitor and subsequent knockdown of RUNX1. **g** Expression of RUNX1, HK1, SLC2A1 was measured by western blot after transfection with miR-30d inhibitor and subsequent knockdown of RUNX1. **h**–**l** Cell proliferation measured by CCK8 assay, colony formation assay, cell migration by transwell assay, cell invasion by transwell assay, tumor angiogenesis by capillary tube formation assay was performed after transfection with miR-30d inhibitor and subsequent knockdown of RUNX1. Scale bars = 100 μm. **m** Panc-1 cells stably expressing miR-30d or joint treatment with RUNX1 overexpression were injected into nude mice. The growth curve was plotted (right) and stripped tumors are shown (left). **n** The tumors were extracted and weighted after 16 days. **o** Sections of tumor were stained with ki-67, MMP9 and CD31 antibodies by immunohistochemical assay. Scale bars = 100 μm.

To further determinate whether RUNX1 promoted glycolysis, it was found that knockdown of RUNX1 or overexpression of RUNX1 could suppress or increase lactate secretion, glucose uptake and intracellular ATP levels compared with controls (Fig. 5a and Supplementary Fig. 5a). Analysis of multiple datasets using a Multiple Experiment Matrix (MEM) revealed positive association between RUNX1 and SLC2A1/HK1 (Supplementary Fig. 5b), and knockdown of RUNX1 downregulated SLC2A1 and HK1 expression at protein levels (Fig. 5b, c), while RUNX1 overexpression upregulated SLC2A1 and HK1 protein levels (Supplementary Fig. 5c). These results confirm that RUNX1 is a regulator of SLC2A1 and HK1.

As a transcription factor, RUNX1 controls various target genes by transcriptional regulation. Therefore, it is reasonable to speculate that RUNX1 regulates SLC2A1 and HK1 expression by binding directly to their promoters. ChIP assay showed that RUNX1 was able to bind to the DNA fragments #1 and #3 of the HK1 promoter and the DNA fragment #1of the SLC2A1 promoter, respectively (Fig. 5d). There were three putative RUNX1 binding sites in the HK1 promoter and 4 putative RUNX1 binding sites in the SLC2A1 promoter. We cloned the wild-type HK1/SLC2A1 promoters (WT) or HK1/SLC2A1 promoter binding site mutants into a luciferase reporter plasmid, and demonstrated knockdown of RUNX1 decreased the luciferase activity of the WT reporters, while HK1 site 2 mutant (MT2) and SLC2A1 site 1 mutant (MT1) were not responsive to RUNX1 knockdown (Fig. 5e and Supplementary Fig. 5d), demonstrating that RUNX1 binds to binding site 2 of HK1 and binding site 1 of SLC2A1 to regulate glycolysis.

To further investigate whether miR-30d functions through targeting RUNX1, we demonstrated that lactate secretion, glucose uptake and intracellular ATP levels, and SLC2A1/HK1 could be regulated by targeting RUNX1 directly via miR-30d (Fig. 5f, g, and Supplementary Fig. 5e, f). Furthermore, miR-30d knockdown promoted cell growth, migration, invasion, and tumor angiogenesis, while RUNX1 knockdown attenuated these processes (Fig. 5h, l); miR-30d mimics inhibited cell growth, cell migration, cell invasion, and tumor angiogenesis, whereas RUNX1 cotransfection rescued these processes (Supplementary Fig. 5g–k). In addition, in vivo assay showed that overexpression of RUNX1 alone significantly promoted tumor growth compared with the control group (Supplementary Fig. 5l, m). Moreover, overexpression of RUNX1 markedly attenuated the ability of miR-30d to inhibit PDAC growth in vivo (Fig. 5m–o). Taken together, miR-30d plays a tumor-suppressive role in resisting pancreatic tumorigenesis via a selective target loss of HK1 and SLC2A1-regulator RUNX1, subsequently resulting in inhibition of the Warburg effect.

### m^6^A-mediated miR-30d biogenesis via YTHDC1-induced promotion of pri-miR-30d degradation

The potential mechanisms responsible for the downregulation of miR-30d in pancreatic cancer were investigated. To this end, we first demonstrated that neither histone acetylation nor DNA methylation was involved in the downregulation of miR-30d in PDAC cells (Supplementary Fig. 6a, b).

**Fig. 6:**
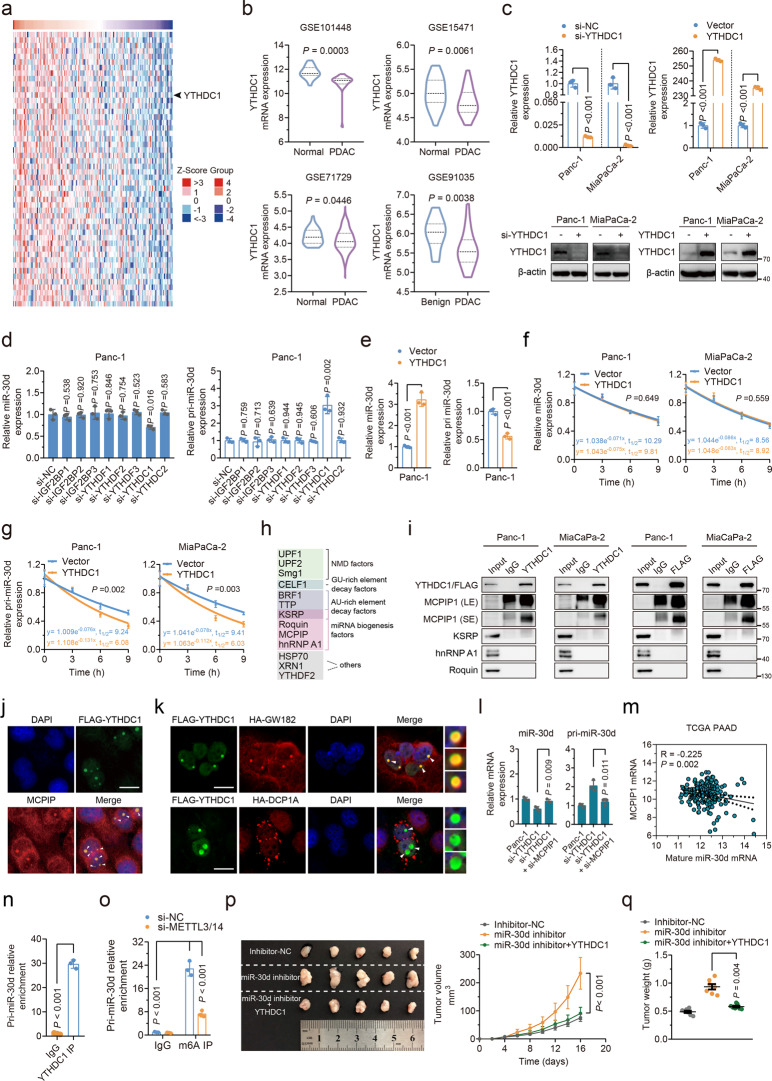
m^6^A-mediated upregulation of miR-30d via YTHDC1-induced regulation of mRNA stability. **a** Heatmap showing the top 50 highly associated genes with miR-30d ranked by Pearson’s correlation index using LinkedOmics database. **b** Expression analysis of YTHDC1 in PDAC and normal pancreatic tissue samples using four independent GEO datasets (GSE101488, GSE15471, GSE71729, and GSE91035). **c** mRNA expression level by qRT–PCR and protein expression level by western blot of YTHDC1 were evaluated in two cell lines after knockdown or overexpression of YTHDC1. **d** miR-30d expression or pri-miR-30d was evaluated by qRT–PCR after knockdown of indicated 8 genes in Panc-1 cells. **e** miR-30d or pri-miR-30d expression was evaluated by qRT–PCR after overexpression of YTHDC1 in Panc-1 cells. **f**, **g** qRT-PCR analysis of the mRNA stability of miR-30d and pri-miR-30d after overexpression of YTHDC1 and subsequent treatment with 8 μg/mL actinomycin D at the indicated time points. **h** Identification of mRNA decay proteins. NMD nonsense-mediated mRNA decay. **i** Co-immunoprecipitation and western blotting showing the binding of miRNA biogenesis regulatory factors mRNA decay factors with YTHDC1 or FLAG-tagged YTHDC1 in Panc-1 and MiaCaPa-2 cells, representative of three independent experiments. LE long exposure, SE short exposure. **j** Immunofluorescence showing the colocalization of endogenous MCPIP and FLAG-YTHDC1 in Panc-1 cells. **k** Co-localization of FLAG-YTHDC1 proteins with HA-GW182 or HA-DCP1A in Panc-1 cells. Arrowheads indicate co-localization in nuclear granules. Scale bars, 20 μ m. Images are representative of three independent experiments. **l** Expression of miR-30d and pri-miR-30d was measured by qRT–PCR after transfection with si-YTHDC1 and subsequent knockdown of MCPIP1. **m** Association between MCPIP1 expression and mature miR-30d expression was analyzed by Pearson’s correlation analysis in TCGA cohort. **n** YTHDC1 interacts with pri-miR-30d, as determined by RIP assay using YTHDC1 antibody. **o** Enrichment of pri-miR-30d by m^6^A antibody MeRIP-qPCR analysis in Panc-1 cells with and without knockdown of METTL3/14. **p** Panc-1 cells stably expressing miR-30d overexpression or joint treatment with YTHDC1 overexpression were injected into nude mice. The growth curve was plotted (right) and stripped tumors are shown (left). **q** The tumors were extracted and weighted after 16 days.

Interestingly, we found YTHDC1, a ‘reader’ of m^6^A modification, was significantly positively associated with miR-30d expression (Fig. 6a). Since it has been reported that m^6^A modification can regulate the biogenesis of miRNAs [28, 29], we focused on YTHDC1. We first found that YTHDC1 mRNA expression was downregulated in cancer tissues compared with nontumor tissues (Fig. 6b), and there was a positive association between YTHDC1 and miR-30d (Supplementary Fig. 6c) and a negative association between YTHDC1 and RUNX1, SLC2A1, HK1 (Supplementary Fig. 6d). Of note, patients with PDAC with higher YTHDC1 expression had a favorable prognosis compared those with a lower YTHDC1 expression (Supplementary Fig. 6e, f). Furthermore, we detected miR-30d mRNA expression in cells with knockdown of corresponding readers and found miR-30d could only be downregulated after knockdown of YTHDC1 (Fig. 6c, d; Supplementary Fig. 6g). Intriguingly, only si-YTHDC1 significantly increased the expression of pri-miR-30d in PDAC cells (Fig. 6c, d; Supplementary Fig. 6g). We next overexpressed YTHDC1 in PDAC cells (Fig. 6c) and detected mature miR-30d and pri-miR-30d expression by qRT-PCR. Consistent with results of knockdown of YTHDC1, overexpression of YTHDC1 downregulated pri-miR-30d and upregulated mature miR-30d (Fig. 6e; Supplementary Fig. 6h). The results that knockdown of YTHDC1 could decrease the mature miR-30d while increase pri-miR-30d indicated that YTHDC1 might regulate the miRNA biogenesis by m^6^A modification. This was confirmed that YTHDC1 had no effect on the half-life of mature-miR-30d (Fig. 6f) while significantly decreased the half-life of pri-miR-30d (Fig. 6g) in both PDAC cell lines. Meanwhile, depletion of YTHDC1 had no effect on the half-life of mature-miR-30d (Supplementary Fig. 6i) while significantly increased the half-life of pri-miR-30d (Supplementary Fig. 7a) in both PDAC cell lines. Consistently, half-life of mature-miR-30d and pri-miR-30d was not influenced after knockdown of the other seven readers (Supplementary Fig. 6j, k). Similarly, YTHDC1 depletion had no significant effect on the decay or stability of pri-miR-200c and mature miR-200c-3p (Supplementary Fig. 7b), which has been widely reported to be involved in the occurrence and development of pancreatic cancer [30–33].

**Fig. 7:**
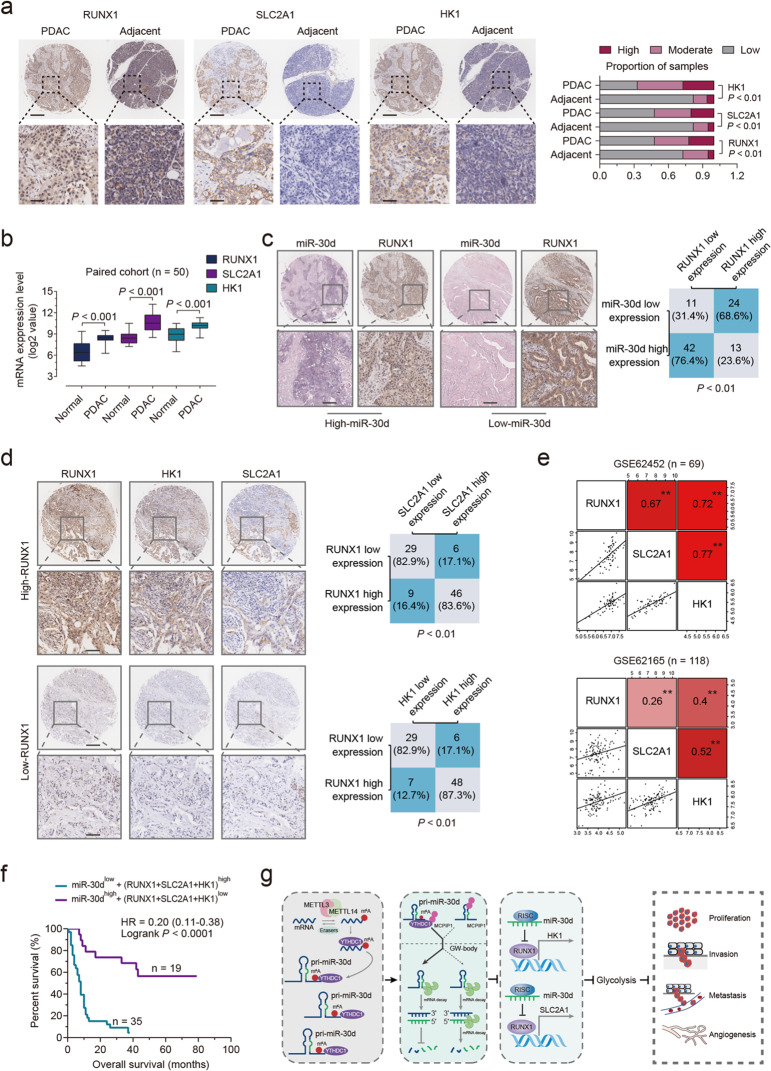
Clinical significance of miR-30d/RUNX1/SLC2A1/HK1 axis in PDAC patients. **a** Representative immunohistochemical images (left) of RUNX1, HK1 and SLC2A1 in PDAC tissues and adjacent nontumor tissues using IHC analysis and statistical analysis of proportion of high, moderate, and low staining in PDAC and paired adjacent tissues (right) in the Shanghai cohort. Scale Bars, up: 200 μm; down: 50 μm. **b** Expression analysis of RUNX1/HK1/SLC2A1 in PDAC and matching normal pancreatic tissue samples in the GSE102238 dataset. **c** Representative in situ hybridization images of miR-30d and immunohistochemical images of RUNX1 expression in PDAC with miR-30d low expression and high expression (left), and statistical analysis of PDAC tissues under different staining conditions (right). Scale Bars, up: 200 μm; down: 50 μm**. d** Representative immunohistochemical images of RUNX1 and HK1/SLC2A1 expression in PDAC with RUNX1 low expression and high expression (left), and statistical analysis of PDAC tissues under different staining conditions (right). Scale Bars, up: 200 μm; down: 50 μm. **e** Correlation between RUNX1 expression and HK1/SLC2A1 expression of pancreatic tissues in 2 GEO datasets (GSE62452, GSE62165). The square in the upper right corner demonstrates the Pearson correlation value between the indicated genes with blue indicating negative correlation and red indicating positive correlation. The square in the lower left corner shows the scatterplot matrix fitted trend line for indicated genes. **, correlation is significant at the 0.01 level. **f** Kaplan–Meier analysis of overall survival between PDAC patients with miR-30d low expression and three highly expressed markers RUNX1/SLC2A1/HK1 and those with miR-30d high expression and three lowly expressed markers RUNX1/SLC2A1/HK1 in the PDAC TMA. **g** Schematic diagram of the relationship among YTHDC1, miR-30d, RUNX1, HK1, SLC2A1, glycolysis metabolism, and pancreatic tumorigenesis.

To identify co-factors of YTHDC1 that may enhance degradation of mRNA targets, we pulled down the complexes of YTHDC1 immunoprecipitation. It was confirmed that only MCPIP1 could bind to endogenous or ectopically expressed YTHDC1 in PDAC cells, rather than others mRNA decay factors that also have been reported to be able to regulate mRNA decay in diverse mechanisms (Fig. 6h, i; Supplementary Fig. 7c). Moreover, co-localization of MCPIP1 and YTHDC1 was observed in nuclear granules (Fig. 6j). Notably, it was well reported that MCPIP1 is a ribonuclease that acts as a broad suppressor of miRNA activity and biogenesis [34]. We then asked in which cellular compartment YTHDC1 localizes and functions. Immunocytochemical analysis showed that YTHDC1 localized mainly in the nucleus as dot-like structures (Supplementary Fig. 7d), which was highly likely in GW-bodies. Unlike processing bodies (P-bodies), which are predominantly cytoplasmic, GW-bodies are present in both nuclei and the cytoplasm [35]. Importantly, GW-bodies are composed of non-translating RNAs [35]. YTHDC1 well co-localized with GW-body maker GW182, however, no colocalization was identified between YTHDC1 and P-body marker DCP1A (Fig. 7k), which further reveals the role of YTHDC1 in target pri-mRNA degradation. In addition, knockdown of MCPIP1 caused substantial upregulation of miR-30d, whereas strongly reduced pri-miR-30d expression (Supplementary Fig. 7e). Moreover, depletion of MCPIP1 abolished the regulating effect of miR-30d and pri-miR-30d by knockdown of YTHDC1 (Fig. 7l). Further analysis of TCGA PAAD data revealed MCPIP1 expression was negatively correlated with mature miR-30d (Fig. 7m). The upregulation of MCPIP1 was associated with poor overall survival and disease-free survival of PDAC patients (Supplementary Fig. 7f), which is consistent with previous reports that elevated MCPIP1 expression is associated with poor survival in others tumors [36]. Thus, our data indicate that YTHDC1 can promote pri-miR-30d degradation and subsequent processing to mature miRNA through antagonizing termination of miRNA biogenesis of MCPIP1.

The dynamic regulation of m^6^A modification is likely determined by the functional interplay among the m^6^A methyltransferases and demethylases [37]. Therefore, we performed RNA immunoprecipitation (RIP) or m^6^A immunoprecipitation (MeRIP) qPCR assays and identified that there was significant enrichment of pri-miR-30d in pellets precipitated with YTHDC1 or m^6^A antibodies (Fig. 6n, o). To determine whether the expression of YTHDC1 targets is also affected by the level of cellular m^6^A, we knocked down the expression of main writers and erasers, and found that only depletion of m^6^A methyltransferase METTL3 and METTL14 could significantly suppress the expression of mature miR-30d by nearly 50% (Supplementary Fig. 7g). However, we found knockdown of main methyltransferases and demethylases, including depletion of METTL3 or METTL14, had no significant effect on RUNX1 expression in PDAC cells, which is consistent with Simon et al.’s throughput sequencing results [38] (Supplementary Fig. 7h, i). Moreover, the enrichment of pri-miR-30d in pellet precipitated with m^6^A antibody was significantly decreased after lowering m^6^A level through knockdown of METTL3/14 (Fig. 6o; Supplementary Fig. 7j). The above results indicate that the m^6^A mark acts as a key post-transcriptional modification that may mainly drive the initiation of miR-30d biogenesis without regulating the expression of RUNX1.

We demonstrated that knockdown of METTL3 or METTL14 could not suppress or increase lactate secretion, glucose uptake and intracellular ATP levels (Supplementary Fig. 7k). However, we found that knockdown of YTHDC1 could markedly promote lactate secretion, glucose uptake and intracellular ATP levels compared with controls (Supplementary Fig. 7l). As one of metabolic stress conditions, we found that lactic acid had no significant effect on m^6^A levels (Supplementary Fig. 7m). Moreover, YTHDC1 alone significantly reduced Panc-1 tumor growth and tumor weight in xenograft mouse tumor models (Supplementary Fig. 7n), and vice versa (Supplementary Fig. 7o). Furthermore, ectopic expression of YTHDC1 partially rescued the miR-30d knockdown-induced tumor burden (Fig. 6p, q). The results taken together indicated that only YTHDC1, as a key regulator of miR-30d biogenesis, might regulate aerobic glycolysis through altering miR-30d processing.

### Clinical significance of miR-30d/RUNX1/SLC2A1/HK1 axis in PDAC patients

Immunohistochemistry (IHC) staining showed that the levels of RUNX1, SLC2A1, and HK1 were higher in PDAC tissues than those in normal tissues (Fig. 7a), and the mRNA expression profiles of RUNX1/SLC2A1/ HK1 axis were more highly expressed in the PDAC tissue than those in normal tissue in the seven GEO cohorts (Supplementary Fig. 8a). The mRNA expression profile of RUNX1/SLC2A1/ HK1 axis in PDAC was further confirmed in another four paired PDAC cohorts (Fig. 7b; Supplementary Fig. 8b). Meanwhile, patients with PDAC had a higher expression of RUNX1/SLC2A1/ HK1 axis than patients with benign neoplasms (Supplementary Fig. 8c).

With samples from the 90 patients, we showed that expression level of miR-30d was reversely associated with the expression level of RUNX1 (Fig. 7c), and that samples expressing high RUNX1 tended to have high expression of SLC2A1 and HK1 (Fig. 7d). Meanwhile, samples with high expression of miR-30d also tended to have low expression of SLC2A1 and HK1 (Supplementary Fig. 8d). We also found positive association between RUNX1 expression and SLC2A1 or HK1 expression in another 6 GEO cohorts (Fig. 7e; Supplementary Fig. 8e). We next assessed the association between RUNX1, glycolysis components, and overall survival after tumor resection in the PDAC TMA and another 2 cohorts. This analysis showed that elevated expression of RUNX1, HK1 or SLC2A1 in PDAC tissues predicted robustly shorter overall survival intervals in the PDAC TMA, TCGA, and GSE62452 cohorts (Supplementary Fig. 8f). Furthermore, shortest overall times or disease-free times were detected in those patients with low expression of miR-30d combined with three highly expressed markers RUNX1/SLC2A1/HK1 in the PDAC TMA and TCGA cohort (Fig. 7f; Supplementary Fig. 8g). From these observations, we conclude that elevated expression levels of miR-30d and the lowered expression of its downstream target genes may identify PDAC patients with good prognosis. So far, we have proved that YTHDC1-induced miR-30d may function as a tumor suppressor gene by negatively regulating RUNX1 and its downstream glycolytic genes including HK1, and SLC2A1 (Fig. 7g).

## Discussion

In this study, we presented evidence that miR-30d functions as a tumor-suppressive molecule, directly or indirectly targeting key glycolytic genes independent of oncogenic transcription factors HIF1a and MYC, and rewiring the cellular metabolism to reduce glycolysis and repress PDAC tumorigenesis.

The increase in glycolysis is mainly caused by increased expression of enzymes and transporters involved in glucose uptake, lactate production, and lactate secretion [39]. In this paper, we discovered that miR-30d was significantly negatively related to the expression level of genes related to glucose metabolism. Subsequently, validation and functional studies suggested that SLC2A1 (GLUT1) and HK1 were the critical downstream genes of miR-30d in PDAC. To clarify the molecular mechanisms responsible for the inhibitory effect of miR-30d on aerobic glycolysis in PDAC cells, we predicted bioinformatically, and biologically validated RUNX1 as the only target transcription factor on regulating SLC2A1 and HK1 expression. Our data indicate that miR-30d is a key tumor-suppressive molecule for regulation of the Warburg effect and also indicate a causal role for miR-30d/RUNX1 axis in glycolysis regulation.

In this study, we linked the abnormal m^6^A modifications in pri-miR-30d to the development and progression of PDAC through glycolysis signal transduction. Previous studies have shown that mRNA transcripts with m^6^A modifications are often regulated by YTHs or IGF2BPs as the direct m^6^A readers [15]. Among these readers, YTHDC1 is a nuclear m^6^A reader and shares almost completely overlapping sites with m^6^A in nuclear RNAs. YTHDC1 recruits the serine and arginine-rich splicing factor 3 (SRSF3), restricts exon-skipping factor SRSF10 binding, and promotes exon inclusion [24]. YTHDC1 also plays a key role in the processing of pre-mRNA in the nucleus of oocytes by interacting with the pre-mRNA 3′-end processing factors CPSF6, SRSF3, and SRSF7 [40].

Herein, we verified that depletion of YTHDC1, not other readers, makes for the global reduction of miR-30d and concomitant accumulation of unprocessed pri-miR-30d in PDAC cells. Additionally, we found that overexpression of YTHDC1 substantially shortened the half-life of pri-miR-30d, suggesting that YTHDC1-mediated pri-miR-30d mRNA decay was at least in part due to the decreased stability of its mRNA transcript. In present study, the mRNA decaying function of YTHDC1 was supported by its co-factor, MCPIP1. MCPIP1 is a ribonuclease that acts as a broad suppressor of miRNA activity and biogenesis [41]. MCPIP1 competes with Dicer1 in miRNA processing and has been described to suppress miRNA biosynthesis via cleavage of the terminal loops of pre-miRNAs [34]. In pancreatic cancer cells, MCPIP1 decreases the expression of miR-200 family members through counteracting Dicer1 in miRNA maturation process [42]. Interestingly, we showed that MCPIP1 was co-localized with YTHDC1 in GW-bodies, which are locations for non-translating RNAs fate decision. Our findings suggest that MCPIP1 could be antagonized by YTHDC1 to promote m^6^A-containing pri-miR-30d to degradation and facilitate miRNA biogenesis. One previous article found that s-adenosylmethionine synthesis is regulated by selective m^6^A methylation and mRNA degradation involving METTL16 and YTHDC1 [23]. A recent study also discovered that YTHDC1 promotes PTEN mRNA degradation to increase Akt phosphorylation, thus facilitating neuronal survival after ischemia [21]. In addition, YTHDC1 could regulate certain non-coding RNA accumulation in a cell-type independent way [22]. Furthermore, we found that METTL3/14 depletion significantly decreased the amount of pri-miR-30d modified by m^6^A. However, we demonstrated that knockdown of METTL3 or METTL14 had no influence on lactate secretion, glucose uptake and intracellular ATP levels. Notably, we found that knockdown of YTHDC1 could markedly regulate biological behavior associated with aerobic glycolysis. These results indicated that the m^6^A marks enhance the recognition of pri-miR-30d by YTHDC1 and the subsequent processing to mature miRNA, which support our study that YTHDC1-mediated biogenesis of miR-30d suppresses glycolysis in PDAC.

In summary, our results suggest that one of the tumor-suppressive effects of increased expression of miR-30d is to inhibit RUNX1 and inactivate the aerobic glycolysis signaling, forming a vigorous YTHDC1-miR-30d-RUNX1-SLC2A1/HK1 axis to repress PDAC occurrence and progression.

## Materials and methods

### Cell culture and treatment

Human pancreatic cancer cell lines Panc-1 and MiaPaCa-2 were purchased from American Type Culture Collection and cultured in Dulbecco’s Modified Eagle’s Medium, supplemented with 10% fetal bovine serum (Gibco) with a 37 °C water-saturated 5% CO_2_ atmosphere. The other PDAC cell lines, including HPNE, AsPC-1, BxPC-3, Capan-1, Capan-2, CFPAC-1, HPAC and SW1990, were a gift from Dr. Lingye Tao from Renji hospital. The miR-30d mimics, miR-30d inhibitor, siRNAs against human RUNX1, HIF1α, and MYC, or controls were transfected into the pancreatic cells using the DharmaFECT transfection reagent (Thermo Scientific Dharmacon Inc., USA). The sequences of the siRNAs were listed in Supplementary Table 1. The plasmids for RUNX1, YTHDC1, HA-GW182 and HA-DCP1A were constructed by Generay Technologies (Shanghai, China) and transfected into the pancreatic cells using the FuGENE transfection reagent (Life Technologies, USA). The lentivirus control, lentiviral vectors expressing miR-30d were constructed by Shanghai OBiO medical biotechnology company, Shanghai, China.

### RNA-Seq and microarray processing

The RNA-Seq data and additional patient information were downloaded from public TCGA (http://cancergenome.nih.gov/) PAAD data repositories. Count-based differential expression pipeline for mRNA-seq or miRNA-seq data were analyzed using R based package edge. The microarray data and additional patient information were downloaded from GEO repository with the accession numbers GSE24279, GSE31568, GSE41369, GSE53325, GSE71533, GSE62452, GSE60987, GSE59856, GSE102238, GSE101488, GSE32676, GSE60979, GSE62165, GSE71729, GSE71989, GSE28735, GSE15471, GSE16515, GSE91035. Data were preprocessed with ‘impute’ package when needed. For finding differentially expressed microRNAs, data were divided into two patterns including pancreatic cancer and normal pancreatic tissue. Differentially expressed miRNAs were acquired by ‘limma’ package in R 3.2.1. Fold changes ≥2 and False Discovery Rate (FDR) ≤ 0.05 were considered statistically significant.

Comparison of miR-30d expression levels in TCGA PADD was conducted on the basis of expression values (log2) obtained by RNA-sequencing analysis (RNA-seq) of miR-30d in the provisional TCGA datasets. Genes that are significantly correlated with miR-30d were determined based on a Spearman correlation analysis (*P* < 0.05). These selected genes were then subjected to GSEA. GSEA was performed to investigate the potential biological pathways involved in PDAC pathogenesis via miR-30d. FDR of 0.25 was established as cut-off for the identification of biologically relevant genes. The gene sets with FDR more than 0.25 were considered enriched between the classes under comparison. The gene sets collection (c2.all.v4.0.symbols.gmt) from the Molecular Signatures Database-MsigDB (http://www.broad.mit.edu/gsea/msigdb/index.jsp) was used for the enrichment analysis.

### Cell proliferation

Cell proliferation was evaluated by Cell Counting Kit-8 (Dojindo, Japan). Cells with indicated treatment were seeded onto 96-well plates at a density of 3 × 10^3^ cells per well with addition of CCK-8 (10 μl per well) to the wells at the specified time point. The reaction product was measured at 450 nm in a microplate reader after incubating for 2 h.

### Colony formation assay

Treated cells were harvested, and 500 cells were seeded into 6-well culture plates. After 7–10 days of incubation, the cells were fixed with 4% paraformaldehyde, stained with 0.1% crystal violet, washed with water and dried. Finally, the colonies were counted. Only those cell clusters containing more than 50 cells under a microscope were considered as colonies.

### Cell cycle test

Cell cycle test was performed using PI/RNase staining buffer (BD Biosciences, Lake Franklin, NJ, USA) on the BD LSR Fortessa instrument according to the manufacturer’s instructions. Flowjo software was used to analyze the percentage of cells at different phases of the cell cycle.

### Apoptosis assay

Apoptosis assay was performed using Annexin V-FITC/PI apoptosis detection kit (BD Biosciences, Lake Franklin, NJ, USA) on the BD LSR Fortessa instrument according to the manufacturer’s instructions. Flowjo software was used to analyze the percentages of apoptotic cells.

### Matrigel migration and invasion assay

Matrigel Migration assay was performed using 24-well transwells (8-μm pore size; Minipore). Matrigel invasion assay was performed using 24-well transwells (8-μm pore size; Minipore) precoated with Matrigel (BD Biosciences, San Jose, CA). 1 × 10^5^ pretreated cells suspended in 200 μL non-FBS cell culture medium were added to the upper chamber and 600 μL culture medium containing 20% FBS was added to the lower chamber and cultured at 37 °C. At indicated time, cells that had migrated onto the lower surface of the membrane were fixed with 4% paraformaldehyde for 15 min and stained with crystal violet for 20 min. After a further wash with PBS, the membranes were air-dried and cell number on the membrane was counted under light microscopy at ×400 magnifications. The number of migrated cells was expressed as the average of five randomly selected fields.

### Tube formation assay

HUVECs at a density of 2 × 10^4^ per well were seeded to matrigel-coated (BD Biosciences, San Jose, CA) 24-well plates with condition media of Panc-1 cells. After incubation at 37 °C for 12 h, cells were stained with Calcein AM (Abcam, Cambridgeshire, UK) and the endothelial tubule formation was photographed using an inverted confocal microscope. Cumulative tube length and branch points were quantified using the Image J software.

### Lactate production assay

L-Lactate Assay kit (Colorimetric) was used to measure the lactate production (Abcam, Cambridgeshire, UK) according to the manufacturer’s protocols. The transfected cells were plated into 96-well cell culture plates and incubated at 37 °C overnight. After starvation for 2 h, the supernatant was collected for measurement of lactate production. The lactate production levels were measured at 450 nm in a microplate reader.

### Glucose uptake assay

Glucose Uptake Colorimetric Assay Kit (Abcam, Cambridgeshire, UK) was used to determine glucose uptake according to the manufacturer’s protocols. 1 × 10^4^ pretreated cells were plated into 96-well cell culture plates and incubated at 37 °C overnight. Next day cells were starved for glucose for 2 h. After incubating with 100 μl Krebs-Ringer-Phosphate-HEPES for 40 min, 10 μl 10 mM 2-DG was injected into each well and incubated for 20 min. Cells were then collected with extraction buffer and used for determination of glucose uptake. The glucose uptake was measured by OD at 412 nm wavelength.

### ATP production assay

ATP Assay Kit (Abcam, Cambridgeshire, UK) was used to measure cellular ATP contents according to the manufacturer’s protocol. Briefly, 100 μl of the cell lysate was mixed with 100 μl of ATP reaction mix and incubated for 30 min. Absorbance was measured by OD at 570 nm wavelength.

### RNA analysis, extraction, and quantitative real-time PCR

Total RNA was extracted by TRIzol reagent (Invitrogen), and 1 μg of total RNA was reverse transcribed using the PrimeScript RT Reagent Kit (Perfect Real-Time; Takara) and was measured using a real-time quantitative PCR system. The amplified transcript level of each specific gene was normalized to ACTB. For miRNAs, 0.5 µg of the total RNA was reverse transcribed into cDNA using a specific miRNA stem loop primer. The amplified transcript level of each specific gene was normalized to U6. The primers were provided by Shenggong Company and shown in Supplementary Table 1.

### RNA stability assay

Cells with indicated transfection were subsequently treated with Actinomycin D for 0 h, 3 h, 6 h, and 9 h at a final concentration of 3 μM. Total RNA was extracted, and real-time PCR was conducted to quantify the relative level of pri-miR-30d or miR-30d mRNA. The degradation rate and half-life of pri-miR-30d or miR-30d mRNA were estimated.

### Western blots

Protein extracts were resolved through 8–15% SDS-PAGE, transferred to PVDF membranes, and probed with primary antibodies. Peroxidase-conjugated anti-mouse or rabbit antibody (Kangcheng, China) was used as secondary antibody and the antigen-antibody reaction was visualized by enhanced chemiluminescence assay. The following commercial antibodies were used: anti-human YTHDC1 (Cat. no. 14392-1-AP; PROTEINTECH), HK1 (Cat. no. sc-46695; Santa Cruz), SLC2A1 (Cat. no. sc-377228; Santa Cruz), ENO1 (Cat. no. sc-100812; Santa Cruz), RUNX1 (Cat. no. ab23980; Abcam), ACTB (Cat. no. KC-5A08; Kangcheng), MYC (Cat. no. 5605; CST), HIF1α (Cat. no. 14179S; CST), FLAG (F1804, Sigma), FLAG (Cat. no. 2368; CST), MCPIP1 (Cat. no. sc-515275; Santa Cruz), KSRP (Cat. no. ab140684; Abcam), hnRNP A1(Cat. no. sc-32301; Santa Cruz), Roquin (Cat. no. ab70196; Abcam), UPF1 (Cat. no. ab109363; Abcam), UPF2 (Cat. no. sc-398424; Santa Cruz), Smg1 (Cat. no. 4993; CST), CELF1 (Cat. no. sc-20003; Santa Cruz), BRF1 (Cat. no. sc-81405; Santa Cruz), TTP (Cat. no. sc-374305; Santa Cruz), HSP70 (Cat. no. 4872; CST), XRN1 (Cat. no. sc-165985; Santa Cruz) and YTHDF2 (Cat. no. 24744-1-AP; PROTEINTECH).

### Immunofluorescence

Cells growing on the coverslips were washed with PBS, fixed in 4% paraformaldehyde for 15 min and treated with 0.3% Triton-x in PBS for 5 min. Cells were blocked with 3% BSA for 1 h at room temperature and incubated with the specific primary antibodies and accordingly dye-conjugated secondary antibody. Finally, cells were counterstained with DAPI (Vector Laboratories, Bulingame, CA). The images were captured using a laser-scanning confocal microscope (LSM-710, Zeiss, Germany).

### ChIP

A ChIP assay kit (Millipore, New Bedford, MA) was used according to the manufacturer’s instructions. Panc-1 cells cultured in a 10-cm dish were fixed with 16% formaldehyde to crosslink proteins to DNA. Chromatin was extracted from the cells using anti-RUNX1 antibody (Cat. no. ab23980; Abcam) and crosslinked DNA was sheared into 250–500 bp fragments. IgG was used as negative. PCR primers (Supplementary Table 1) were used to detect putative RUNX1-binding fragments.

### RIP

RIP assays were conducted using the Magna RIP Kit (Millipore, New Bedford, MA) according to the manufacturer’s protocols. Cells were prepared using RIP lysis buffer and the RNA-protein complexes were immunoprecipitated using anti-YTHDC1 antibody (PROTEINTECH, Rosemont, IL) and normal rabbit IgG. The co-precipitated RNAs were purified using phenol:chloroform:isoamyl alcohol and subjected to reverse transcription-PCR or real-time PCR analysis. A control amplification was carried out on the input RNA before immunoprecipitation.

### MeRIP

The specific anti-m^6^A antibody (Synaptic Systems, Goettingen, Germany) was applied for MeRIP. Anti-m^6^A antibody was pre-bound to Protein G magnetic beads in reaction buffer for 30 min. The fragmented mRNA was incubated with m^6^A-antibody-bound protein G magnetic beads at 4 °C for 1 h and washed with low salt reaction buffer and high salt reaction buffer. m^6^A-antibody-bound RNA was extracted from the Dynabeads using Buffer RLT (Qiagen, Hilden, German) and further incubated with Dynabeads MyOne Silane (Life Technologies, West Palm Beach, FL). The RNA and Dynabeads mixture were precipitated with 100% ethanol, washed with 70% ethanol and then re-suspend with nuclease-free water. The supernatant was carefully collected after the beads were pulled to the side of the tube by a magnetic field. Real-time PCR was carried following m^6^A-immunoprecipitation to quantify the changes of m^6^A methylation of a certain target gene.

### RNA m^6^A dot blot assays

The two fold serial dilution of poly(A)1 RNAs (2000 ng) was performed, and they were then spotted onto a nylon membrane (GE Healthcare). The membranes were then UV crosslinked, blocked, and incubated with m^6^A antibody (ABclonal, A19841). Then HRP-conjugated goat anti-rabbit IgG (Santa Cruz Biotechnology) was added to the blots for 1 h at room temperature and the membrane was then developed with Thermo ECL SuperSignal Western Blotting Detection Reagent (Thermo Fisher Scientific, Waltham, MA).

### Co-IP

50 µl suspended IP Matrix (Santa Cruz, USA), 1 µg normal rabbit IgG or IP antibodies and 500 µl PBS were incubated at 4 °C on a rotator for at least one hour. The mixture was centrifuged and washed two times and then the supernatants were then discarded carefully. Cells that transfected for 48 h were lysed and transferred to the pelleted matrix, incubating at 4 °C on a rotator overnight. After incubation, the matrix was centrifuged and washed for five times. SDS-PAGE sample loading buffer was added to the immunoprecipitates followed by boiling for 10 min at 100 °C. The IP proteins and the input samples were detected by western blot.

### Luciferase assay

To investigate the RUNX1 3ʹ UTR activity, three plasmids were designed and constructed (GENEray): pGL3-RUNX1 3′ UTR wild-type (WT) plasmid, pGL3-RUNX1 3ʹ UTR miR-30d mut1 (MT1) plasmid, pGL3-RUNX1 3ʹ UTR miR-30d mut2 (MT2) plasmid and pGL3-RUNX1 3ʹ UTR miR-30d mut 1 + 2 (MT1 + 2) plasmid. To explore RUNX1 effect on the SLC2A1 and HK1 promoter transcriptional activity, pGL3-SLC2A1 promoter wild-type (WT) plasmid with relevant mut-type (MT) plasmid and pGL3-HK1 wild-type promoter (WT) plasmid with relevant mut-type (MT) plasmid were designed and constructed (GENEray Company, Shanghai, China). The reporter plasmids and the miR-30d mimics, miR-30d inhibitor, relevant siRNAs were cotransferred into cells. Luciferase activity was measured using a FLUOstar device (Omega Engineering, Deckenpfronn, Germany), with the Dual-Luciferase reporter assay system (Promega). Transfection efficiency was normalized by dividing the luciferase activity of the construct by the corresponding Renilla luciferase activity.

### In vivo xenograft model

To illustrate the effect of miR-30d on tumor growth in vivo, 4-week-old male BALB/c nude mice were used in our study. Panc-1 and MiaPaCa-2 cells with miR-30d overexpression or controls were injected into the right flank of mice subcutaneously to establish the PDAC xenograft model. Tumor volume (mm^3^) was estimated by the formula: tumor volume (mm^3^) = short diameter^2^ * long diameter/2. Two weeks after inoculation, tumor-bearing mice were sacrificed and the subcutaneous xenograft tumors were excised and weighted. Finally, all tumors were kept in formalin for Ki-67 (Cat. no. sc-23900; Santa Cruz) staining.

### In vivo metastatic model and bioluminescent imaging

Four-week-old BALB/C nude mice were used and cared for according to the institutional guidelines for animal care. To study the effect of miR-30d on liver metastasis of PDACs, 5 × 10^6^ cells in 50 μl of PBS were injected into the spleens of nude mice (6 mice per group). Anesthetized mice were injected intraperitoneally with D-luciferin (150 mg/kg) every other week and imaged using an IVIS 100 imaging system (Xenogen, CA, USA) 10 min after the injection. The mice were sacrificed and their liver metastases were checked by standard histological examination 8–9 weeks after injection. Finally, all tumors were kept in formalin for further analysis of MMP9, a marker of metastasis (catalog number: sc-12759; Santa Cruz) and CD31, a marker of angiogenesis (catalog number: sc-376764; Santa Cruz).

### IHC Staining and ISH

Tissue microarrays were stained with primary antibodies against HK1 (Cat. no. sc-46695; Santa Cruz), SLC2A1 (Cat. no. sc-377228; Santa Cruz), RUNX1 (Cat. no. ab23980; Abcam). The in situ detection of miR-30d was performed on 6-μm formalin-fixed, paraffin-embedded (FFPE) sections using DIG-labeled miRCURYTM Detection probe (Exiqon, Denmark). Briefly, the slides were hybridized with a probe (LNA-modified and DIG-labeled oligonucleotide; Exiqon) complementary to miR-30d and after incubation with anti–DIG-AP Fab fragments conjugated to alkaline phosphatase. The hybridized probes were then detected by applying nitroblue tetrazolium/5-bromo-4-chloro-3-indolyl phosphate color substrate (Roche) to the slides. Slides were counterstained with VECTOR® nuclear fast red counterstain (VECTOR LABOTATORIES) and analyzed with a Nikon 80i microscope and Nikon NIS-Elements F 2.3 software (Nikon).

### RNA stability assay

PDAC cells with or without corresponding gene knockdown were treated with Actinomycin D for 0 h, 3 h, 6 h, and 9 h at a final concentration of 8 μg/mL. Total RNA was extracted, and real-time PCR was conducted to quantify the relative level of mature miR-30d or pri-miR-30d mRNA. The degradation rate and half-life of miR-30d and pri-miR-30d mRNA were estimated according to the published paper. Briefly, the degradation rate of mRNA (Kdecay) was calculated by the following equation:

$${\mathrm{ln}}\left( {{\mathrm{C/C0}}} \right){\mathrm{ = - Kdecayt}}$$

where *t* is the transcription inhibition time, and C is the mRNA level at the time *t*. C0 is the level of mRNA at 0 h in the equation, which means the mRNA level before decay starts. Thus, the mRNA half-time (t1/2) can be calculated by the equation:

$${\mathrm{In}}\left( {{\mathrm{1/2}}} \right){\mathrm{ = - Kdecayt1/2}}{\mathrm{.}}$$

The difference of mRNA level at the 9 h after administration of Actinomycin D in the corresponding treatment was compared using a two-tailed Student’s *t* test.

### Human tissues microarray

The study protocol was approved by the ethics committee with obtained informed consent from all participants, in accordance with the provisions of the Declaration of Helsinki of 1975. The patient cohort of human PDAC tissue microarray (TMA) containing 90 PDAC specimens and corresponding normal pancreatic tissues without radiotherapy or chemotherapy prior to surgery.

After IHC and ISH staining, tissue microarrays were digitally scanned by the Aperio Scanscope XT automated slide scanner, and the whole field of each tissue spot was obtained for IHC and ISH evaluation. The expression levels of miR-30d, RUNX1, SLC2A1 and HK1 were scored semiquantitatively based on staining intensity and distribution using the immunoreactive score (IRS). Briefly, Immunoreactive score (IRS) = SI (staining intensity) × PP (percentage of positive cells). SI was assigned as: 0 = negative; 1 = weak; 2 = moderate; 3 = strong. PP was defined as 0 = 0%; 1 = 0–25%; 2 = 25–50%; 3 = 50–75%; 4 = 75–100%. For categorization of the continuous IRS values into low and high, we chose a cutoff point for the measurements (range 0–12, cutoff point ≤4 versus >4).

### Statistical analyses

Statistics were performed by GraphPad Prism 8.0 and Excel. Data from at least three independent tests performed in triplicates are presented as the means ± SE. Error bars in the scatterplots and the bar graphs represent SE. For data with normal distribution, ANOVA was employed for statistical analysis when more than two groups were compared, and a two-tailed Student’s *t* test was used when comparison between two groups was performed. Nonparametric Kruskal–Wallis rank sum test was used for continuous variable with non-normally distributed data. Comparison of Kaplan–Meier survival curves was performed with the log-rank test. One representative experimental result was selected to display in present study from three or more independent experiments.

## Supplementary information

Figure S1

Figure S2

Figure S3

Figure S4

Figure S5

Figure S6

Figure S7

Figure S8

Supplementary Table 1

Supplementary Table 2

Supplementary Table 3

Supplementary material

## Data Availability

The detailed procedures of methods, eight figures and three tables are attached.
