# Supplementary figures and images for: YTHDC1-mediated augmentation of miR-30d in repressing pancreatic tumorigenesis via attenuation of RUNX1-induced transcriptional activation of Warburg effect
Source: Cell Death Differ. 2021 May 21;28(11):3105–24. doi: 10.1038/s41418-021-00804-0 (PMC8563797; doi:10.1038/s41418-021-00804-0)

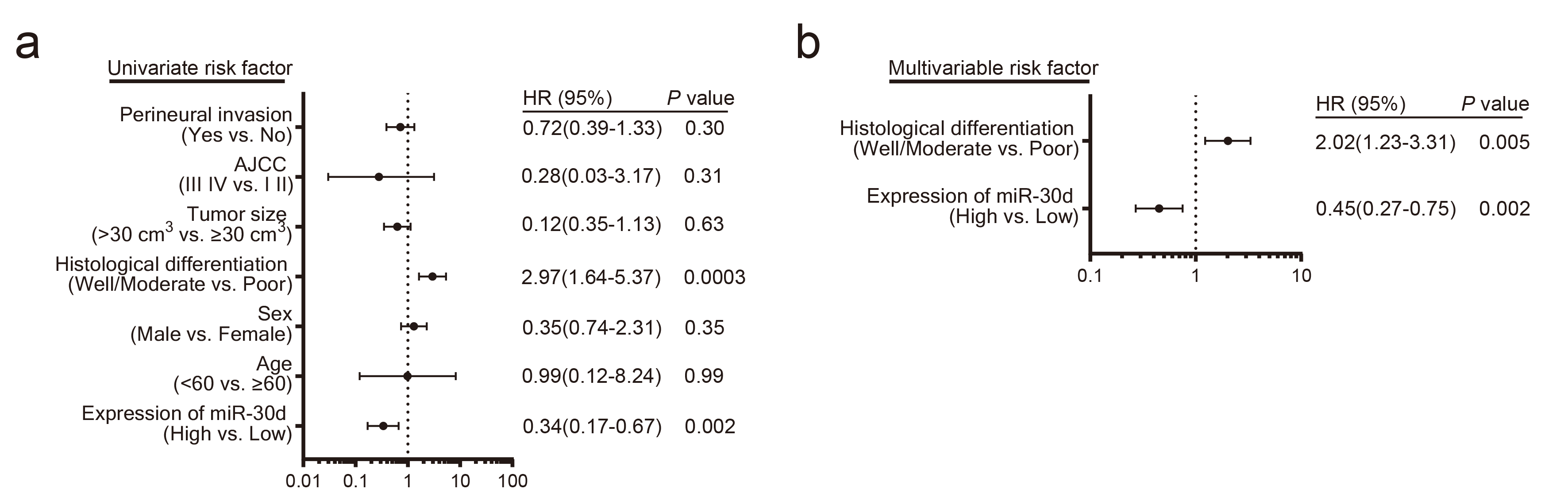

Supplement: Supplementary file 1 — Figure S1 [file 41418_2021_804_MOESM1_ESM.tif]

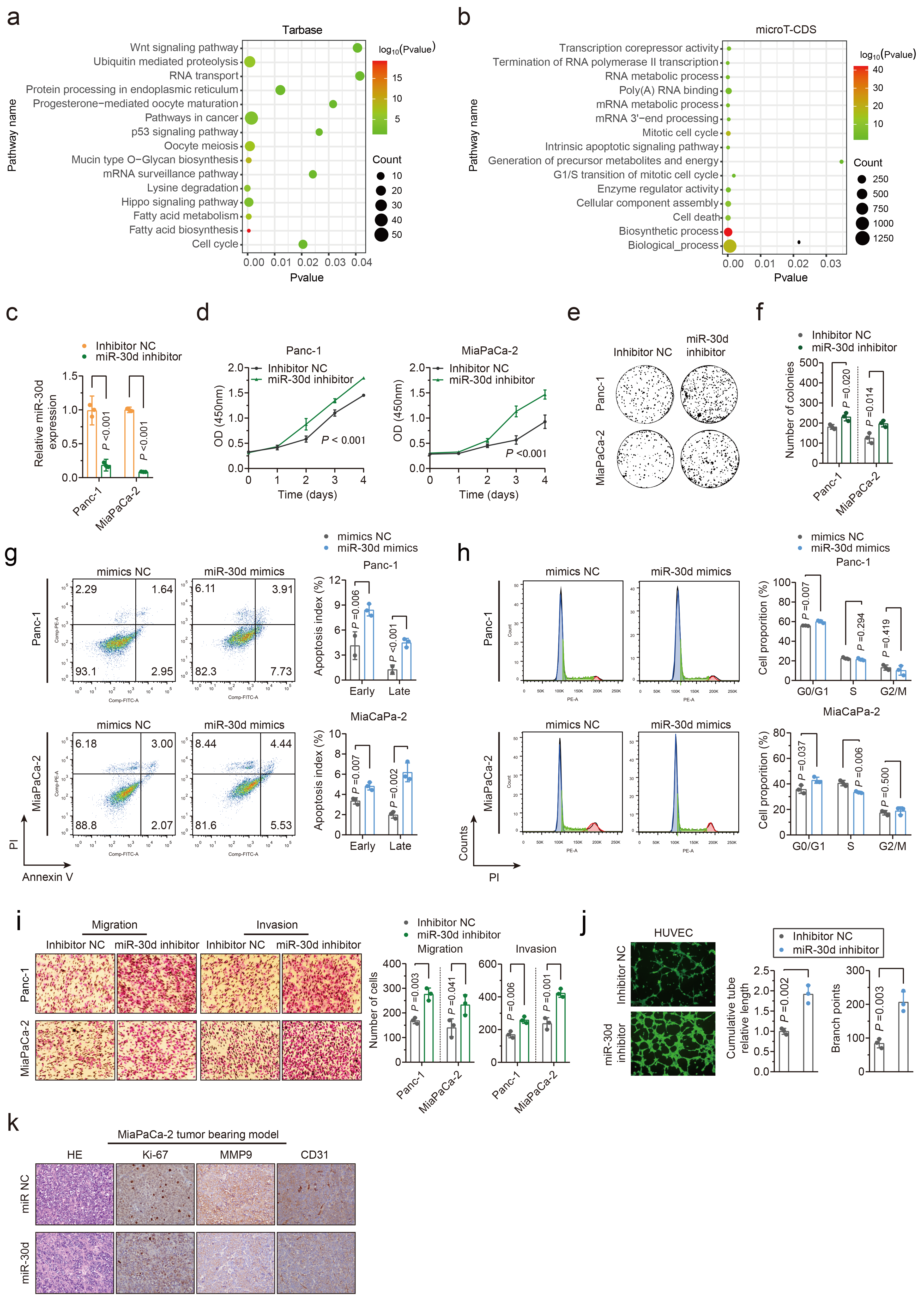

Supplement: Supplementary file 2 — Figure S2 [file 41418_2021_804_MOESM2_ESM.tif]

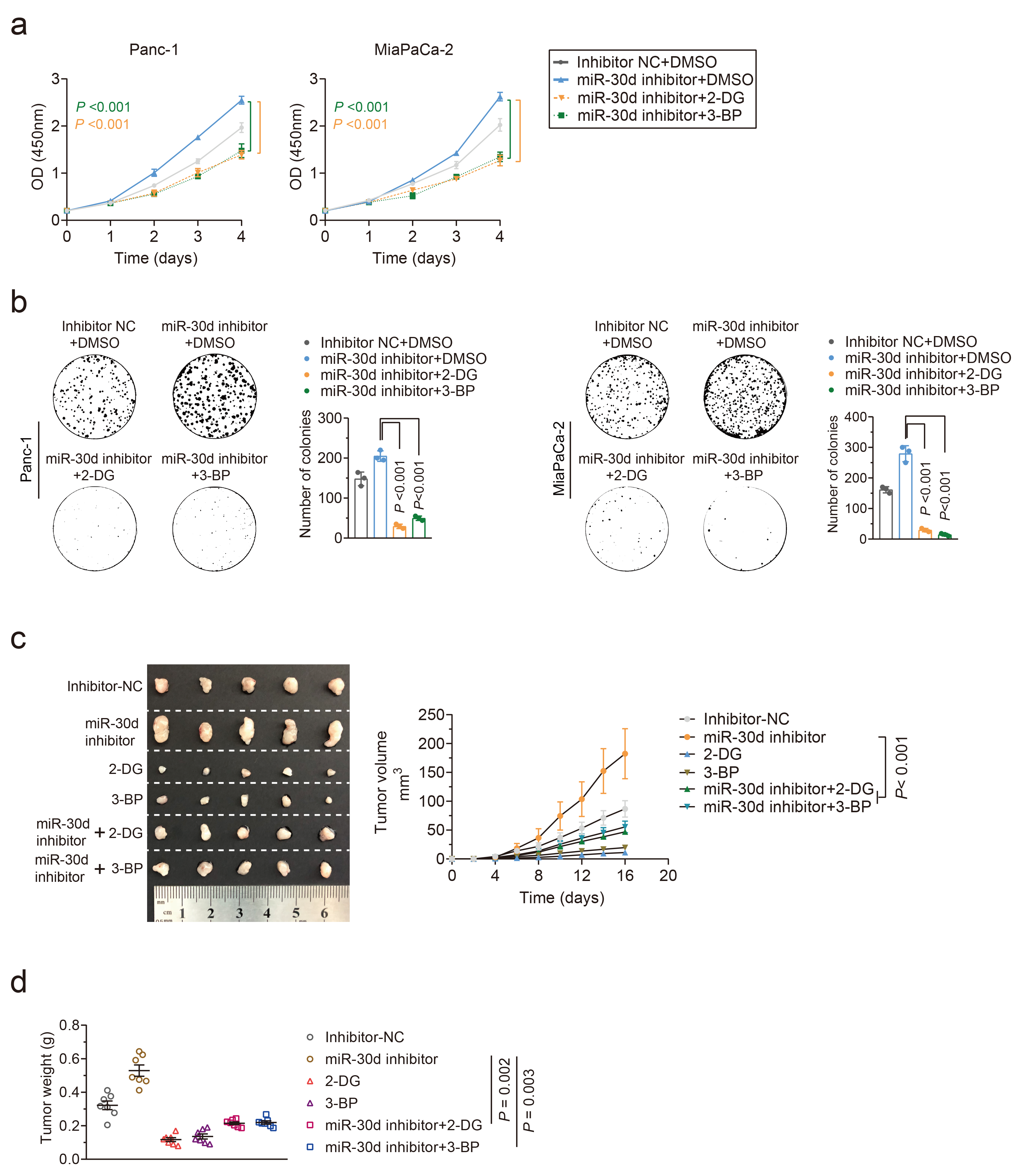

Supplement: Supplementary file 3 — Figure S3 [file 41418_2021_804_MOESM3_ESM.tif]

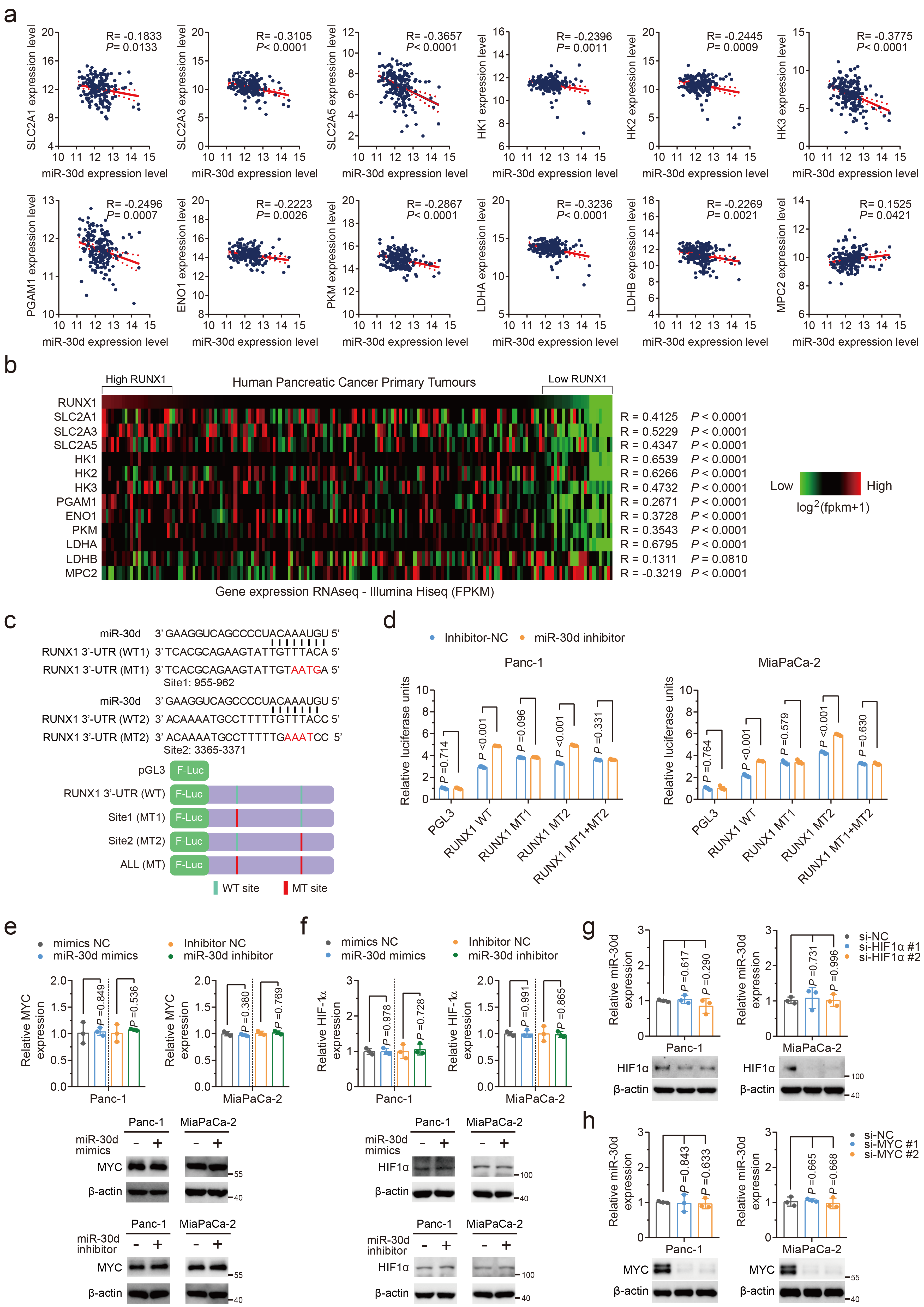

Supplement: Supplementary file 4 — Figure S4 [file 41418_2021_804_MOESM4_ESM.tif]

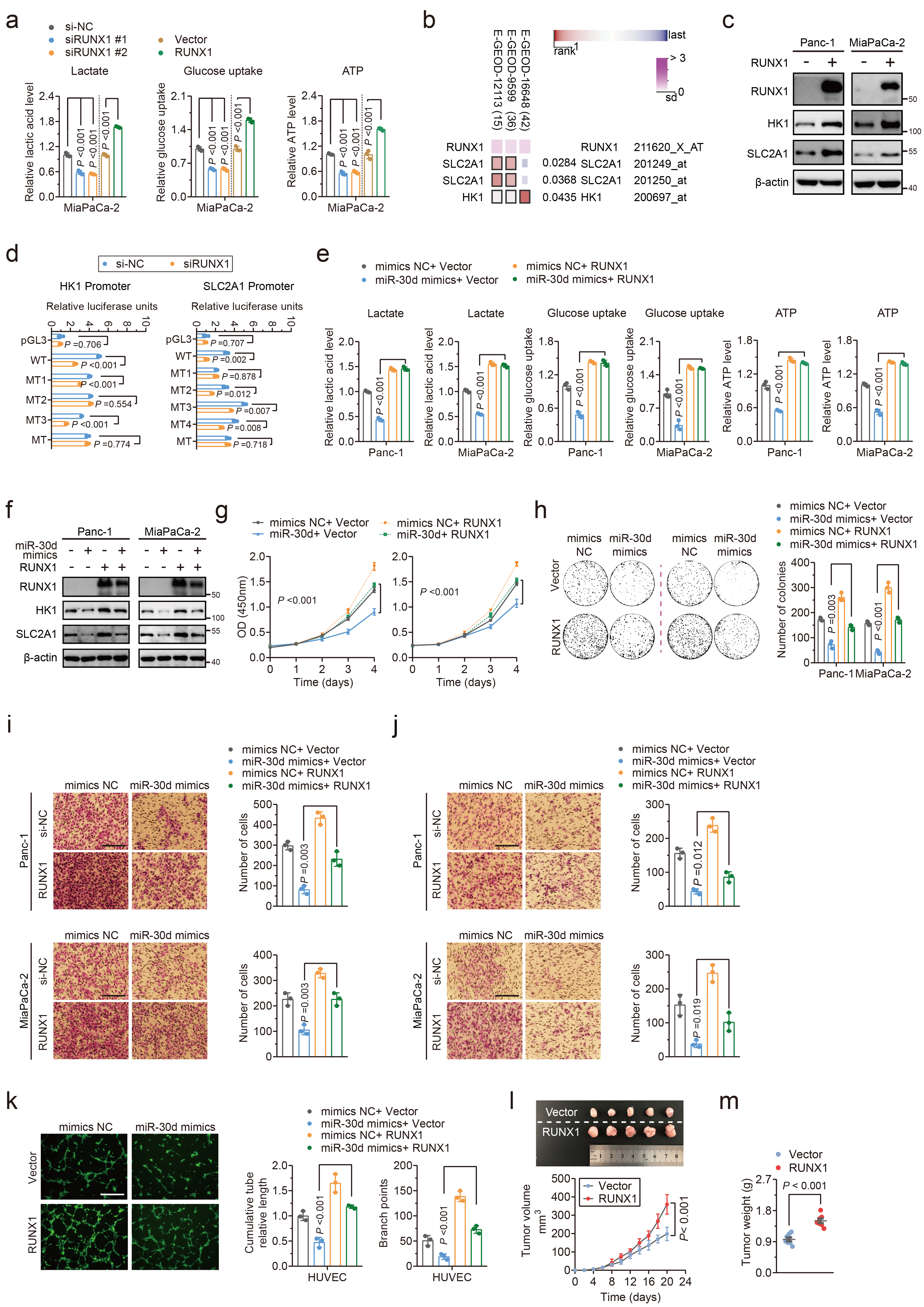

Supplement: Supplementary file 5 — Figure S5 [file 41418_2021_804_MOESM5_ESM.tif]

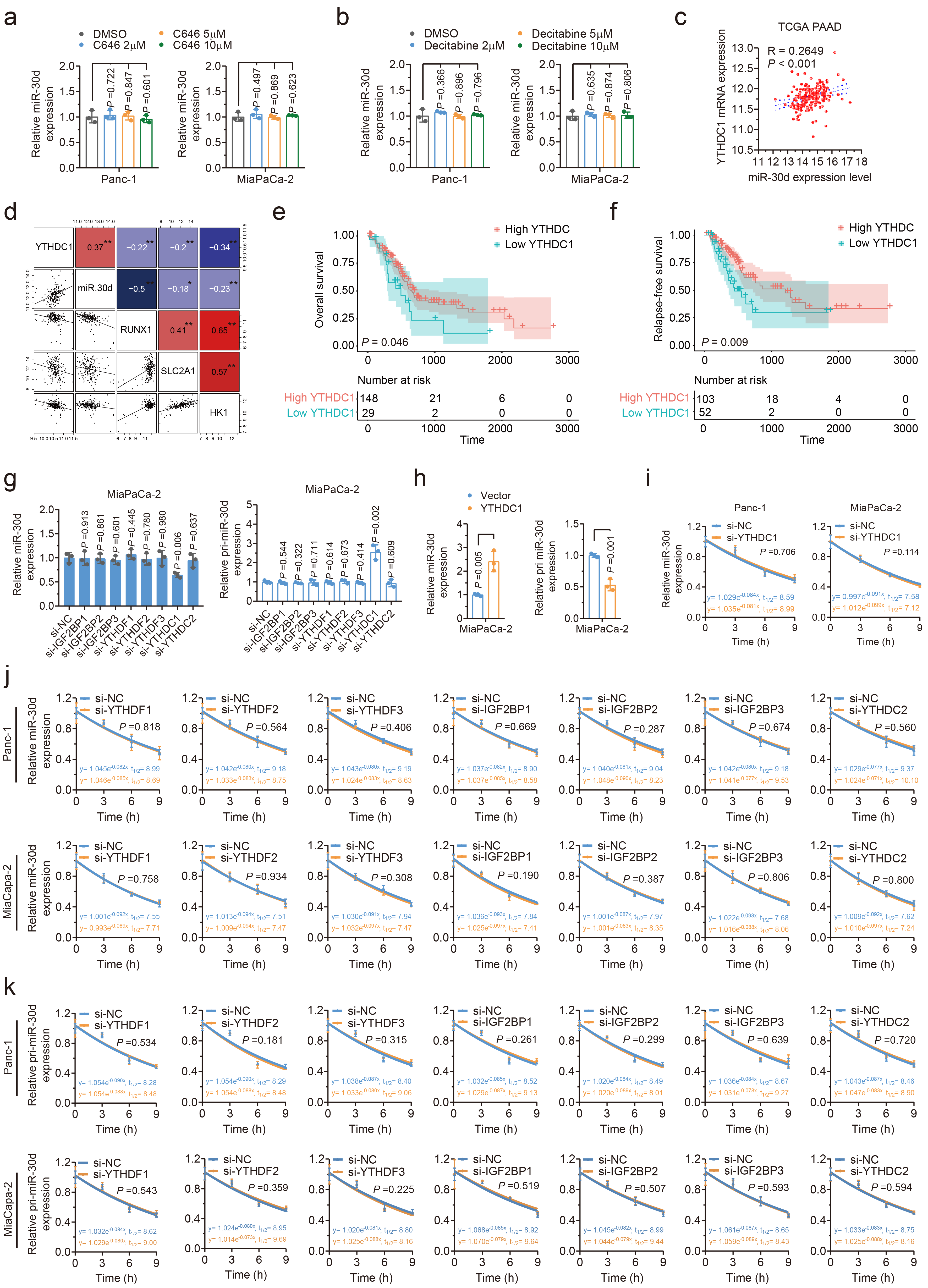

Supplement: Supplementary file 6 — Figure S6 [file 41418_2021_804_MOESM6_ESM.tif]

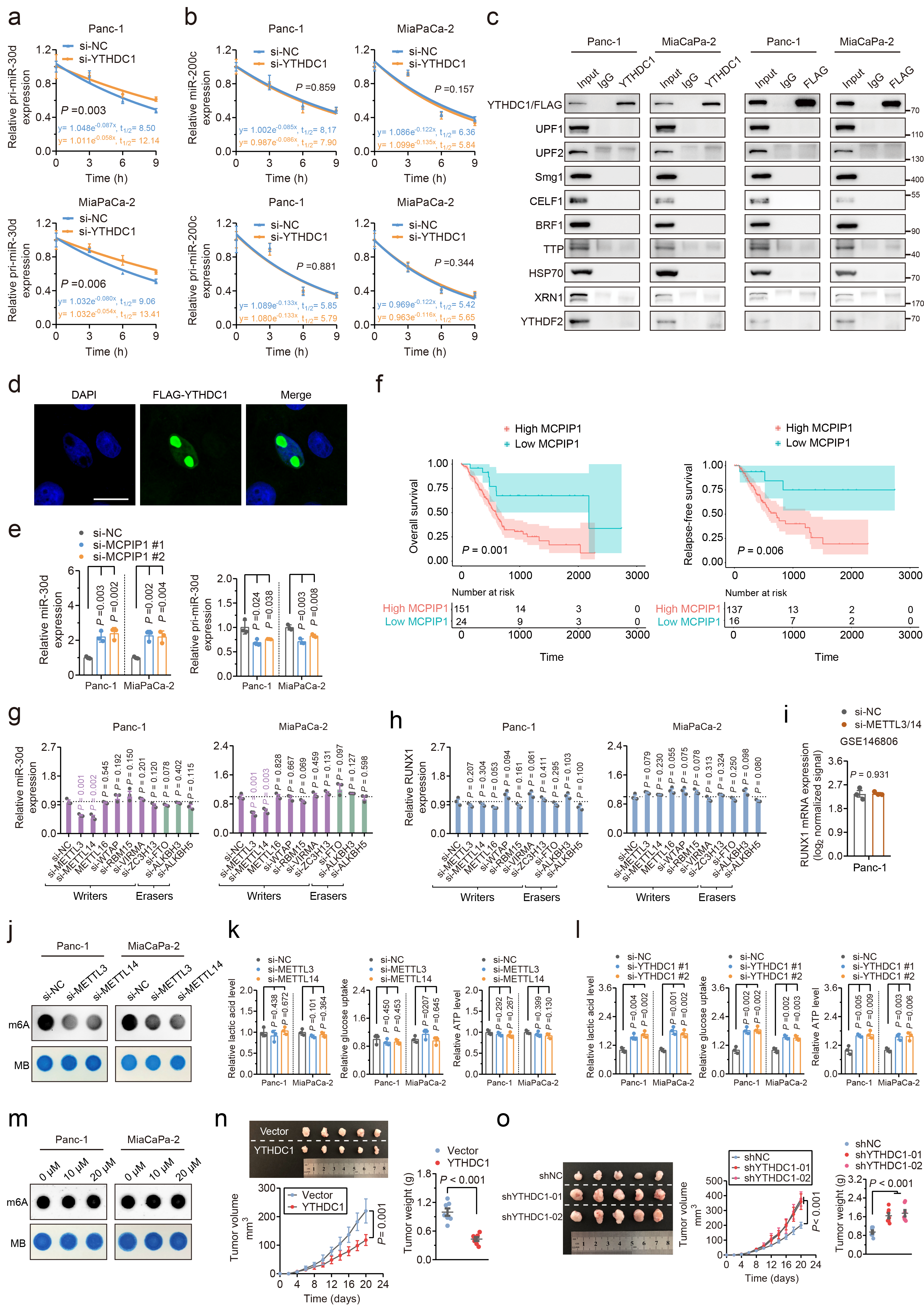

Supplement: Supplementary file 7 — Figure S7 [file 41418_2021_804_MOESM7_ESM.tif]

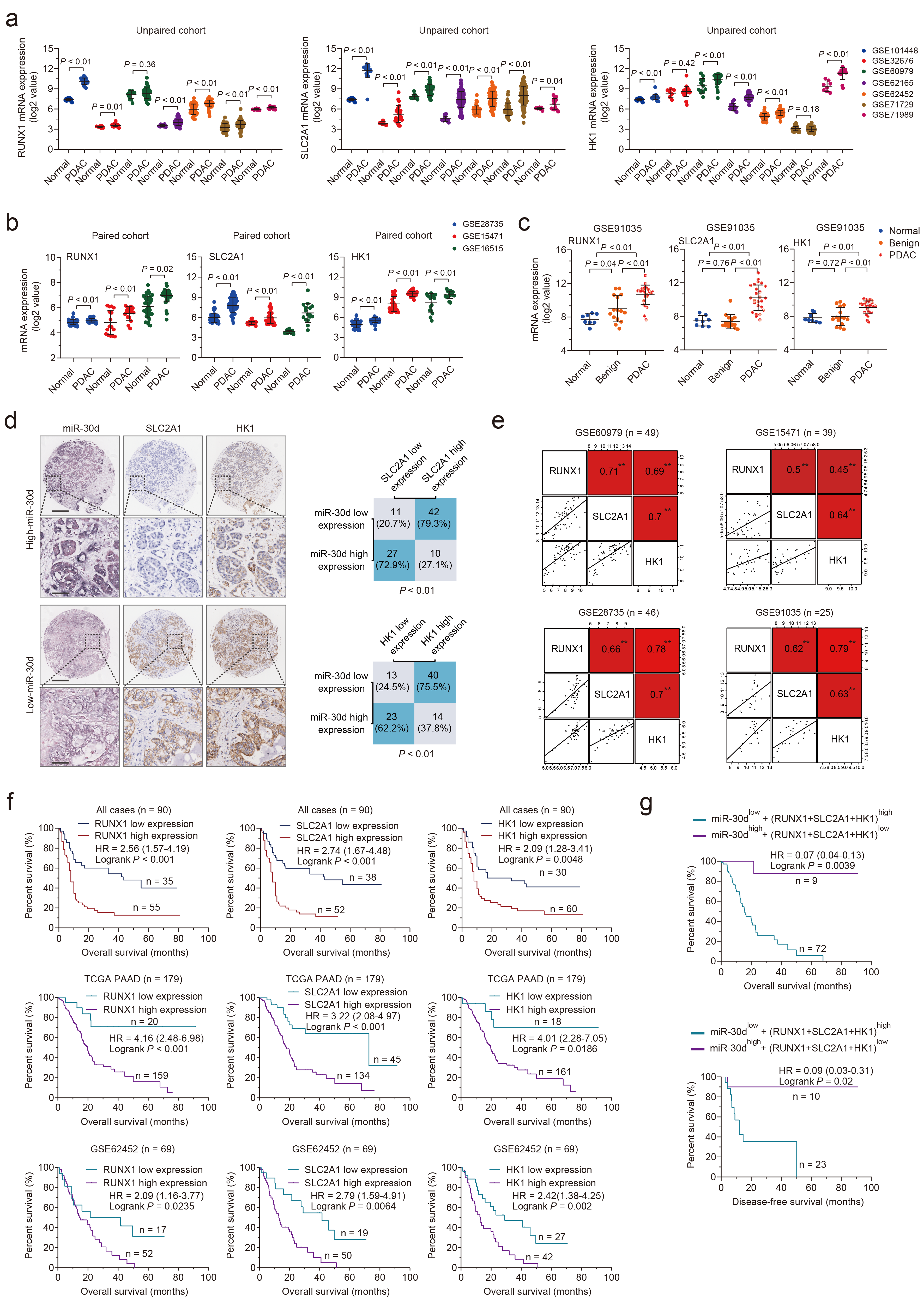

Supplement: Supplementary file 8 — Figure S8 [file 41418_2021_804_MOESM8_ESM.tif]
