# Supplementary material for: YTHDC1-mediated augmentation of miR-30d in repressing pancreatic tumorigenesis via attenuation of RUNX1-induced transcriptional activation of Warburg effect
Source: Cell Death Differ. 2021 May 21;28(11):3105–24. doi: 10.1038/s41418-021-00804-0 (PMC8563797; doi:10.1038/s41418-021-00804-0)
Supplement: Supplementary file 12 — Supplementary material [file 41418_2021_804_MOESM12_ESM.docx]

**Supplementary figure legends**

**Supplementary Fig. 1** **miR-30d expression is correlated with good prognosis in PDAC**. **a** Univariate analysis was performed for identification of risk factors for poor survival of PDAC patients in the PDAC TMA. All the bars correspond to 95% confidence intervals. **b** Multivariable analysis was performed for identification of independent risk factors for poor survival of PDAC patients in the PDAC TMA. All the bars correspond to 95% confidence intervals.

**Supplementary Fig. 2** **miR-30d suppresses tumor proliferation, metastasis, and angiogenesis both *in vitro* and *in vivo***. **a, b** KEGG analysis of miR-30d using DIANA tools based on public Tarbase and microT-CDS datasets. **c** miR-30d expression levels were evaluated using qRT–PCR in indicated cell lines with miR-30d inhibitor. **d** CCK8 assays were performed to determine the cell viability of Panc-1 and MiaPaCa-2 cell lines with miR-30d inhibitor transfection. **e, f** Colony formation assay was performed in two cell lines with miR-30d inhibitor transfection. **g** Flow cytometry was used to evaluate early and late apoptotic percentages of two cell lines with miR-30d mimics transfection. **h** Cell population in each phase was evaluated by flow cytometry in PDAC cell lines with miR-30d mimics transfection. **i** Transwell assays were performed to evaluate the effect of miR-30d on cell migration and invasion in Panc-1 and MiaPaCa-2 cells. Cells were counted under a microscope in five randomly selected fields. Scale bars = 100 μm. **j** Capillary tube formation was performed to evaluate tumor angiogenesis in HUVECs with condition medium of Panc-1 cells with miR-30d inhibitor transfection. Scale bars = 100 μm. **k** Representative immunohistochemical images of Ki-67, MMP9 and CD31 staining from miR-30d and vector subcutaneous xenograft tissues in MiaPaCa-2 tumor bearing model. Scale bars = 100 μm.

**Supplementary Fig. 3** **miR-30d knockdown induced cell growth was attenuated by inhibition of glycolysis**. **a, b** Cell proliferation measured by CCK8 and colony formation assay were performed in Panc-1 and MiaPaCa-2 cells after transfection with inhibitor NC, miR-30d inhibitor, and subsequent treatment with 5 mM 2-DG or 10 μM 3-BP. **c** Panc-1 cells stably expressing inhibitor control and miR-30d inhibitor were injected into nude mice. 2-DG and 3-BP was used as indicated. The growth curve was plotted (right) and stripped tumors are shown (left). **d** The tumors were extracted and weighted after 16 days.

**Supplementary Fig. 4** **miR-30d suppresses glycolysis by inhibiting downstream SLC2A1 and HK1 via directly targeting RUNX1**. **a** Association between miR-30d expression and glucose metabolism-related gene expression was analyzed by Pearson’s correlation analysis in TCGA cohort. **b** Genomic signature of primary human PDAC samples comprising RUNX1 and glucose metabolism-related genes. Samples were sorted dependent on relative RUNX1 expression (high to low). N = 179. **c** Diagram of 3′-untranslated region (3′-UTR) of RUNX1 with putative miR-30d-binding sites. Mutant sequences are indicated in red. **d** Luciferase activity assays were performed to confirm the direct binding efficiency of miR-30d and its putative target RUNX1 with indicated treatment. **e, f** mRNA by qRT-PCR and protein level by western blot of MYC and HIF1α after transfection of miR-30d mimics or inhibitor. **g, h** Expression of miR-30d by qRT-PCR after knockdown of HIF1α or MYC in two cell lines.

**Supplementary Fig. 5** **miR-30d suppresses glycolysis via inhibiting RUNX1 binding to the promoter of SLC2A1 and HK1**. **a** Lactate production, glucose uptake and ATP production were measured by colorimetric analysis in MiaCaPa-2 cells with knockdown and overexpression of RUNX1. **b** RUNX1 and SLC2A1/HK1 are significantly correlated across a range of PDAC datasets according to the MEM output. **c** Protein expression levels of RUNX1, HK1, and SLC2A1 were measured by western blot in two cell lines with overexpression of RUNX1. **d** Luciferase activity in MiaPaCa-2 cells with RUNX1 knockdown following transfection with HK1 or SLC2A1 promoter luciferase reporter vectors. **e** Lactate production, glucose uptake and ATP production were measured by colorimetric analysis after transfection with miR-30d mimics and subsequent overexpression of RUNX1. **f** Expression of RUNX1, HK1, SLC2A1 was measured by western blot after transfection with miR-30d mimics and subsequent overexpression of RUNX1. **g-k** Cell proliferation measured by CCK8 assay, colony formation assay, cell migration by transwell assay, cell invasion by transwell assay, tumor angiogenesis by capillary tube formation assay was performed after transfection with miR-30d mimics and subsequent overexpression of RUNX1. Scale bars = 100 μm. **l** Panc-1 cells stably expressing RUNX1 overexpression were injected into nude mice. The growth curve was plotted (right) and stripped tumors are shown (left). m The tumors were extracted and weighted after 16 days.

**Supplementary Fig. 6** **m^6^A-mediated upregulation of miR-30d via YTHDC1-induced regulation of mRNA stability**. **a, b** Expression of miR-30d by qRT-PCR after treatment of C646 (histone acetyltransferase inhibitor) and decitabine (DNA methylation inhibitor) in two cell lines. **c** Association between YTHDC1 expression and miR-30d expression was analyzed by Pearson’s correlation analysis in TCGA cohort. **d** Correlation between YTHDC1, miR-30d, RUNX1, SLC2A1 and HK1 expression of pancreatic tissues in the TCGA cohort. The square in the upper right corner demonstrates the Pearson correlation value between the indicated genes with blue indicating negative correlation and red indicating positive correlation. The square in the lower left corner shows the scatterplot matrix fitted trend line for indicated genes. **e, f** Kaplan-Meier analysis of the TCGA cohort for YTHDC1 expression. **g** miR-30d expression or pri-miR-30d were evaluated by qRT–PCR after knockdown of indicated 8 genes in MiaPaCa-2 cells. **h** miR-30d or pri-miR-30d expression was evaluated by qRT–PCR after overexpression of YTHDC1 in MiaCaPa-2 cells. **i** qRT-PCR analysis of the mRNA stability of miR-30d after knockdown of YTHDC1 and subsequent treatment with 3 μM actinomycin D at the indicated time points. **j, k** qRT-PCR analysis of the mRNA stability of miR-30d and pri-miR-30d after knockdown of indicated 7 genes and subsequent treatment with 2 μM actinomycin D at the indicated time points.

**Supplementary Fig. 7** **m^6^A-mediated upregulation of miR-30d via YTHDC1-induced regulation of mRNA stability**.

**a** qRT-PCR analysis of the mRNA stability of pri-miR-30d after knockdown of YTHDC1, and subsequently with 8 μg/mL actinomycin D at the indicated time points. **b** qRT-PCR analysis of the mRNA stability of miR-200c-3p and pri-miR-200c after knockdown of YTHDC1, and subsequently with 8 μg/mL actinomycin D at the indicated time points. **c** Co-immunoprecipitation and western blotting showing the binding of mRNA decay factors with YTHDC1 or FLAG-tagged YTHDC1 in Panc-1 and MiaCaPa-2 cells, representative of three independent experiments. **d** Immunocytochemical analysis of overexpressed HA-YTHDC1. Scale bar: 20 mm. **e** miR-30d or pri-miR-30d expression was evaluated by qRT–PCR after depletion of YTHDC1 in Panc-1 and MiaCaPa-2 cells. **f** Kaplan-Meier analysis of the TCGA cohort for MCPIP1 expression. **g, h** miR-30d or RUNX1 expression was evaluated by qRT–PCR after depletion of writers or erasers in Panc-1 and MiaPaCa-2 cells. **i** Differential expression analysis of RUNX1 in Panc-1 cells treated with the knockdown of METTL3/METTL14 and control in GEO dataset (GSE146806). **j** M6A dot blot assays using mRNA of PDAC cells treated with the control and knockdown of METTL3 or METTL14 for 48 h. Methylene blue (MB) staining served as a loading control. **k, l** Lactate production, glucose uptake and ATP production were measured by colorimetric analysis in Panc-1 and MiaCaPa-2 cells with knockdown of METTL3, METTL14 or YTHDC1. **m** M6A dot blot assays using mRNA of PDAC cells treated with increasing lactic acid (0, 10 and 20 mM) for 24 h. Methylene blue (MB) staining served as a loading control. **n, o** Panc-1 cells stably expressing YTHDC1 overexpression or knockdown were injected into nude mice. The growth curve was plotted (right) and stripped tumors are shown (left). The tumors were extracted and weighted after 16 days.

**Supplementary Fig. 8 Clinical significance of miR-30d/RUNX1/SLC2A1/HK1 axis in PDAC patients**. **a** Expression analysis of RUNX1/HK1/SLC2A1 in PDAC and normal pancreatic tissue samples in the seven different GEO datasets (GSE101488, GSE32676, GSE60979, GSE62165, GSE62452, GSE71729, and GSE71989). **b** Expression analysis of RUNX1/HK1/SLC2A1 in PDAC and matching normal pancreatic tissue samples in 3 GEO datasets (GSE28735, GSE15471, GSE16515). **c** Expression analysis of RUNX1/HK1/SLC2A1 in PDAC, benign neoplasms, and normal pancreatic tissue samples in the GSE91035 dataset. **d** Representative *in situ* hybridization images of miR-30d and immunohistochemical images of HK1/SLC2A1 expression in PDAC with miR-30d low expression and high expression (left), and statistical analysis of pancreatic cancer tissues under different staining conditions (right). Scale Bars, up: 200 μm; down: 50 μm. **e** Correlation between RUNX1 expression and HK1/SLC2A1 expression of pancreatic tissues in the 4 GEO datasets (GSE60979, GSE15471, GSE28735, and GSE91035). The square in the upper right corner demonstrates the Pearson correlation value between the indicated genes with blue indicating negative correlation and red indicating positive correlation. The square in the lower left corner shows the scatterplot matrix fitted trend line for indicated genes. **, correlation is significant at the 0.01 level. **f** Kaplan-Meier analysis of overall survival for PDAC patients based on RUNX1, SLC2A1 or HK1 expression in the PDAC TMA, TCGA cohort and GSE62452 cohort. **g** Kaplan-Meier analysis of overall survival or disease-free survival between PDAC patients with miR-30d low expression and three highly expressed markers RUNX1/SLC2A1/HK1 and those with miR-30d high expression and three lowly expressed markers RUNX1/SLC2A1/HK1 in the TCGA cohort.
